# EHreact: Extended Hasse Diagrams for the Extraction and Scoring of Enzymatic Reaction Templates

**DOI:** 10.1021/acs.jcim.1c00921

**Published:** 2021-09-29

**Authors:** Esther Heid, Samuel Goldman, Karthik Sankaranarayanan, Connor W. Coley, Christoph Flamm, William H. Green

**Affiliations:** † Department of Chemical Engineering, Massachusetts Institute of Technology, Cambridge, Massachusetts 02139, United States; ‡ Computational and Systems Biology, Massachusetts Institute of Technology, Cambridge, Massachusetts 02139, United States; § Department of Theoretical Chemistry, University of Vienna, 1090 Vienna, Austria

## Abstract

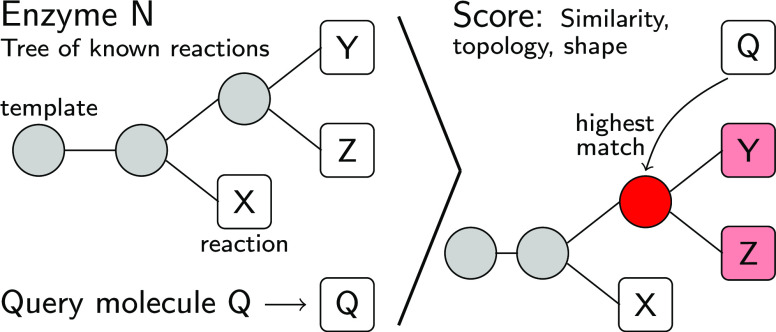

Data-driven computer-aided synthesis planning utilizing organic or biocatalyzed reactions from large databases has gained increasing interest in the last decade, sparking the development of numerous tools to extract, apply, and score general reaction templates. The generation of reaction rules for enzymatic reactions is especially challenging since substrate promiscuity varies between enzymes, causing the optimal levels of rule specificity and optimal number of included atoms to differ between enzymes. This complicates an automated extraction from databases and has promoted the creation of manually curated reaction rule sets. Here, we present EHreact, a purely data-driven open-source software tool, to extract and score reaction rules from sets of reactions known to be catalyzed by an enzyme at appropriate levels of specificity without expert knowledge. EHreact extracts and groups reaction rules into tree-like structures, Hasse diagrams, based on common substructures in the imaginary transition structures. Each diagram can be utilized to output a single or a set of reaction rules, as well as calculate the probability of a new substrate to be processed by the given enzyme by inferring information about the reactive site of the enzyme from the known reactions and their grouping in the template tree. EHreact heuristically predicts the activity of a given enzyme on a new substrate, outperforming current approaches in accuracy and functionality.

## Introduction

Biocatalytic transformations nowadays comprise an ever-expanding toolbox of chemo-, stereo-, and regioselective reactions.^1−7^ The use of enzymes to catalyze reactions has several benefits, such as mild reaction conditions, aqueous media as solvents, compatibility of different reaction steps in multistep syntheses, as well as the reduced need for protecting groups.^3,4,8^ Most enzymes are promiscuous to at least some extent or can be engineered to accept a new substrate, so that the possible range of biocatalyzed transformations is large enough to be of interest to synthetic chemists, as testified by the large number of novel enzymatic cascades for the synthesis of diverse targets that were published in the last decade.^2,3,6,7,9−15^ Enzymatic transformations thus provide a promising and ecofriendly alternative to organic reactions in the synthesis of pharmaceutical intermediates or fine chemicals, among others.^2^

In practice, moderately promiscuous enzymes are often preferred when designing a pathway, where a small amount of activity can be increased via directed evolution.^16^ Enzymes can exhibit both substrate promiscuity and reaction promiscuity,^17^ but within bioretrosynthesis, usually only the former is exploited.^18^ Substrate promiscuity refers to the ability to catalyze the native reaction on a non-native substrate, whereas reaction promiscuity describes the ability to catalyze a non-native reaction. In the following, we will only refer to substrate promiscuity.

To address the challenge of enzymatic synthesis pathway planning, a number of computational tools have been developed for general purpose bioretrosynthesis planning,^18−22^ enzyme selection,^23,24^ metabolic pathway exploration,^18,25^ and reaction rule extraction^26,27^ in recent years. The tools usually extract the catalyzed transformation from a known reaction by identifying the reactive center, coding the changes of atoms and bonds into a reaction rule, and scoring the feasibility of a new substrate undergoing the same transformation on a set of criteria.

Here, a key challenge is to increase the accuracy of the employed scoring functions and thus correctly rank reactions that are anticipated to be feasible higher than reactions that are most likely not catalyzed by the desired enzyme. Whereas some tools consider a reaction feasible if it satisfies a reaction rule at the desired level of specificity,^21,25^ others score the feasibility of a transformation based on chemical similarity to known reactions or substrates via fingerprint vectors.^20,22−24^ However, methods relying on similarity or reaction rule specificity lack the distinction between generalist and selective specialist enzymes, *i.e*., they miss a description of enzyme promiscuity, as pointed out by Jeffryes et al. recently.^28^ By treating each reaction in the database as a separate and independent data point, correlations between known substrates for the same enzymes are lost and with them estimates for enzyme promiscuity and substrate ranges. On the other hand, describing enzymatic promiscuity on the basis of known substrates, as proposed by Nath and Atkins,^29^ can suffer from a lack of data. In fact, a poorly studied enzyme with only a limited set of known substrates might be falsely viewed as highly specific.^28^ However, even an imperfect prediction of promiscuity adds to the accuracy of the predicted reaction feasibility. Furthermore, this limitation becomes less severe as enzymatic reaction databases such as BRENDA,^30^ RHEA,^31^ or KEGG^32^ grow. The ability to pool information across sets of substrates is especially true in the case of BRENDA, where enzymes are reported with a variety of activities on natural and non-natural substrates.

A missing description of enzyme promiscuity furthermore affects the quality of extracted reaction templates. Namely, the specificity of reaction rules extracted from databases of biocatalyzed reactions, *i.e*., the number of atoms included in the template, is usually set by a single user-defined value, treating specific and promiscuous enzymes the same. A few hand-curated sets of enzymatic reaction rules offer enzyme-specific levels of rule generality,^22,24^ but there is currently no method to automatically detect the promiscuity of an enzyme and extract reaction rules accordingly.

We therefore believe that a data-driven approach to extract enzymatic reaction templates at different levels of specificity, as well as to score new queries on criteria beyond fingerprint similarity, is needed, taking into account the estimated promiscuity of an enzyme and the diversity of chemical structures around the reactive center inferred from known substrates.

In this article, we present a novel approach to compute enzymatic reaction templates and predict their applicability on non-natural substrates. We extract reaction templates at levels of specificities imposed by the set of known substrates and arrange them in a tree-like structure (a Hasse diagram of molecule fragments^33^) to allow for an estimation of enzyme promiscuity and substrate range. New substrates are scored on the basis of each template tree by taking into account different measures of overall similarity and diversity, as well as a comparison of the structure of the query substrate to conserved substructures within the known substrates. Our open-source software allows a variety of different queries, including the scoring of a specific reaction, the proposal and ranking of possible reactions on a substrate including regioselectivity and choice of cosubstrates, or scoring of substrates instead of full reactions if the products are unknown. We thus provide a valuable tool to describe and predict enzymatic reactions, which is freely available on Github.^34^

The remainder of this article is organized as follows. The extraction algorithm, as well as details on the employed scoring functions and the preparation of literature data sets, is explained in the Methods section. We then analyze the number of known reactions per enzymes throughout different databases, showcase the template extraction routine on a small example, and compare the performance of the scoring routine regarding activity prediction, regioselectivity, and cosubstrate proposal against fingerprint-based approaches on experimental screening data, as well as reactions from the enzyme database BRENDA in the Results and Discussion section. Concluding remarks are given in the Conclusions section.

## Methods

EHreact is implemented in Python and can be used either as a standalone command line application or imported as a Python package. EHreact uses RDKit to process molecules^35^ and Graphviz^36^ to depict template trees.

### Input Format and Transformation to Imaginary Transition Structure

EHreact can operate in two different template tree generation modes: taking reactions as input (default, recommended) or only the reactants (single substrates).

With standard settings, *i.e*., in reaction mode, EHreact takes a balanced, atom-mapped reaction SMILES as input, which must include explicit hydrogen atoms. If the atom-mapping is not known, it is automatically calculated via the Reaction Decoder Tool^37^ (RDT), a state-of-the-art tool for atom-mapping enzymatic reactions.^38^ In this case, the non-atom-mapped reaction SMILES can be given with or without hydrogens. The accuracy of atom-mappings by RDT for the different enzyme classes in this study is given in the Supporting Information. Since correct atom-mappings are integral to the performance of EHreact, we recommend to precompute mappings via RDT or any other tool and correct them if necessary.

**Figure 1:**
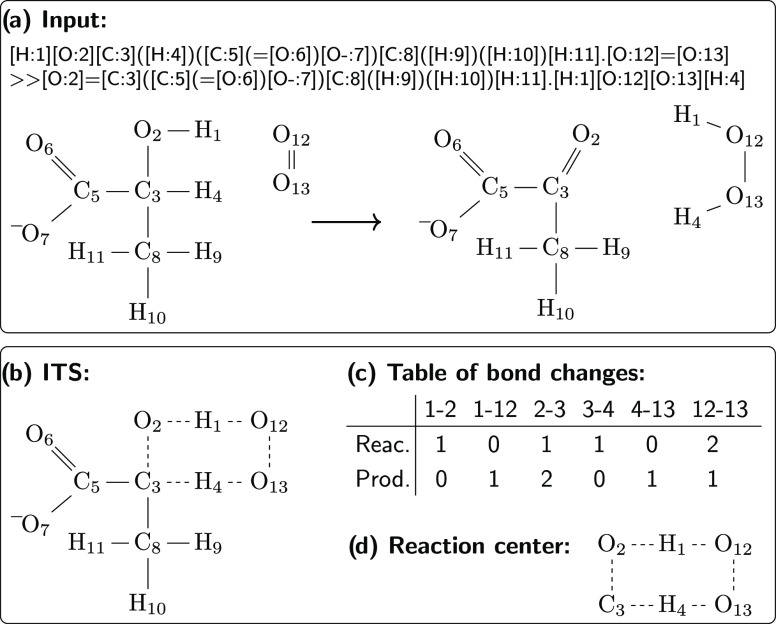
EHreact processes an inputted atom-mapped SMILES string (a, upper box), yielding three outputs (lower box), namely, the respective imaginary transition structures (b), a table of bond changes (c), and the reaction center that comprises only the atoms and bonds undergoing changes or bonds between atoms undergoing changes (d).

Each reaction is then transformed into its imaginary transition structure (ITS), closely related to the condensed graph of reaction, by identifying every atom and bond that changes during the reaction, where we take into account changes in the charge, hybridization, number of radical electrons, aromaticity, or bond number and order into account, following the procedure outlined by Coley et al.^39^ Molecules not contributing atoms to the reaction, for example, reagents, are omitted. The ITS of a reaction is a topological superposition of the reactants and products, where bonds present in only the reactants, only the products, and both reactants and products can appear at the same time.^40^ It therefore describes the graph artificial transition state between reactants and products (but not a true transition state or mechanism). Figure 1 shows an exemplary ITS for the oxidation of lactate via the enzyme lactate oxidase (EC 1.1.3.2). Such imaginary transition structures, although known for decades, have recently attracted increased interest for parsing reaction databases, predicting structure–activity relationships and developing reaction descriptors.^41−43^

We note that the extracted templates in EHreact do not take into account chirality, which is instead treated in the scoring algorithm. Handling stereochemistry on the scoring level instead of the template level has a number of advantages. First, reactions with stereocenters specified only in a part of the inputted reactions do not lead to different templates and thus branching in the template tree if stereoinformation is omitted. This is important because enzyme databases contain entries lacking stereocenters for select reactions, but the correct stereochemistry can usually be inferred from other reactions catalyzed by the same enzyme during scoring. At the template level, missing information on a stereocenter would cause different templates to be extracted for the set of reactions, making a comparison at the scoring stage difficult. Second, not every enzyme is perfectly stereoselective, making it favorable to consider all possible stereoisomers at the template level and filter these stereoisomers later during scoring to account for the selectivity of the enzyme.

In single-substrate mode, EHreact takes SMILES strings as input (e.g., “CC(O)C(=O)[O-]” for lactate), which may be given with or without hydrogen atoms. Since no product is specified in this mode, one can additionally input a seed for the maximum common substructure search in SMILES format to help the algorithm focus on the relevant part of the molecule. For the oxidation of lactate (Figure 1), a meaningful seed would be “C([H])O[H]”, which is simply the secondary alcohol that lactate oxidase transforms to a ketone. If no seed is specified, the algorithm uses the maximum common substructure in all input substrates as seed.

In both reaction and single-substrate modes, multiple seeds or reaction centers can be specified to describe enzymes that catalyze slightly different transformations as long as they are mutually exclusive.

### Template Tree Generation

**Figure 2:**
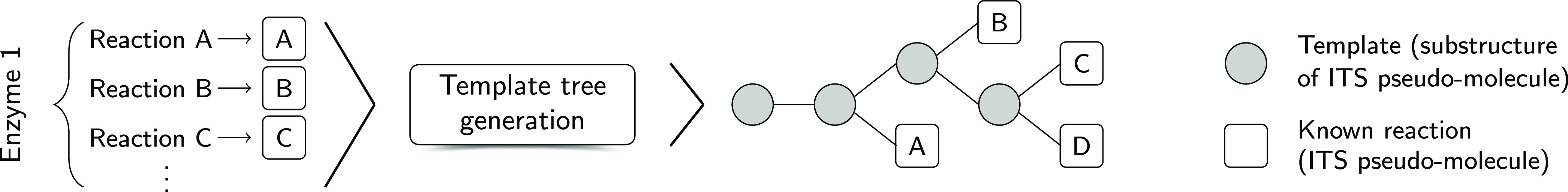
Schematic workflow of the template tree generation: for each enzyme, a list of known reactions is transformed into their respective imaginary transition structures (ITS, white squares) and passed to the template tree generation algorithm. The algorithm outputs a Hasse diagram of the common substructures around the reaction center (reaction templates, gray circles) and the known reactions, which is saved to the file.

After identification of the reactive center (or the seed atoms in substrate mode), the template is expanded in a stepwise manner based on the structures of the known reactions or substrates. In reaction mode, the structures are ITS pseudo-molecules, and the initial atoms comprise the reaction center. In single-substrate mode, the structures are real molecules, namely, the input substrates, and the seed is either given manually or automatically inferred from the maximum common substructure. Since EHreact is per default in reaction mode, we will use the ITS nomenclature in the following. An overview of the template tree generation is given in Figure 2.

**Figure 3:**
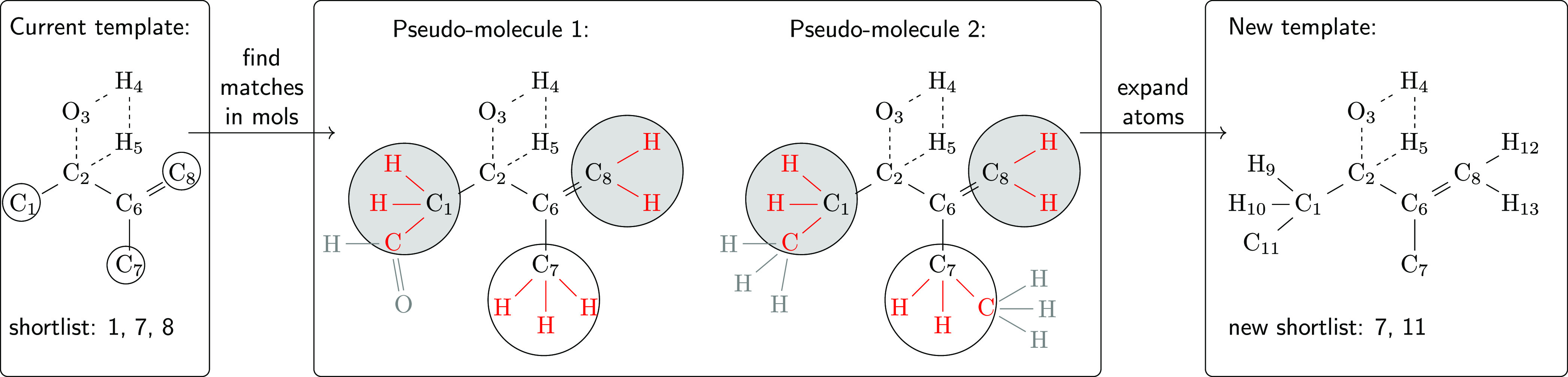
Current template allows atoms 1, 7, and 8 to be extended (not all neighbors specified yet). The neighbors of each shortlisted atom in the template are compared by matching the template to each pseudo-molecule and identifying its neighbors. The algorithm chooses all atoms that have the same neighbors in all pseudo-molecules, here atoms 1 and 8 (highlighted in gray), for extending the template, leading to a new, larger, more specific template.

The algorithm iteratively adds more information to a template, creating a new, more specific template. To this aim, all atoms with unspecified neighbors are shortlisted in the current template. For example, in Figure 1, the reaction center (the current template at the first iteration) can only be expanded at atom C_3_, which is the only atom with unspecified neighbors. If only one atom is shortlisted, the new template is formed from the current template and all neighboring bonds and atoms of the shortlisted atom. If more than one atom is shortlisted, the algorithm searches for a combination of as many atoms as possible that lead to the exact same new, enlarged template. An example of this process is shown in Figure 3 for a set of two known reactions, where, from the shortlisted atoms 1, 7, and 8, only atoms 1 and 8 have the same neighbors in all pseudo-molecules. Thus, the new template is formed from the current template and the neighboring bonds and atoms of only atoms 1 and 8. Multiple matches of the template to a pseudo-molecule may occur, in which case all options are explored, and the match leading to a maximum of mutually expandable atoms across all pseudo-molecules is kept. If multiple combinations are possible, the one including less hydrogen atoms is favored. If only a single pseudo-molecule is known, all shortlisted atoms are expanded, which is similar to a diameter-based extraction of rules.^19,20,26^ If no expansion leads to an applicable template for all pseudo-molecules, all shortlisted atoms are expanded, which leads to a branching in the generated template tree, where multiple new templates emerge from the current template. If an expansion adds an atom in a ring, the full ring is added within a single expansion step.

The generated templates are saved in a template tree, where each new template is attached to its parent template, that is, the template it emerged from. Each template can only have a single parent but one or multiple children. A node in the tree without a child is simply one of the input pseudo-molecules, where all atoms are included in the template, leaving no atoms in the shortlist, and thus no more specific templates that could be attached as children. Mathematically, such a picture is called a Hasse diagram, which is simply a way of ordering and depicting a set of objects using partial orders. Hasse diagrams have been proposed in the literature to be applicable for the ordering of substructures in molecules.^33^ Since we not only save the information of parent and child to the diagram but also a number of additional features, we call the generated template trees “extended Hasse diagrams”.

The diagram is saved to disk in a custom Python class, where all templates are saved as RDKit molecules. Furthermore, EHreact produces a text-based representation of the Hasse diagram, as well as optionally a PNG picture. An example output is given in the Supporting Information.

In summary, if only a single reaction is known, templates are extracted at different diameters from the reaction center, creating a linear Hasse diagram without any branching, but if more than one reaction is known, the algorithm makes use of the mutual structural information between them. In both cases, a number of properties of the template tree and its leaf nodes are then precomputed to speed up the subsequent scoring of a query reaction or substrate. The novelty of this approach is to add atoms and bonds to a reaction center, making use of conserved substructures in all known reactions instead of some predefined radius around the reaction center. Implicitly, we thus assume that conserved substructures indicate the importance of the respective structures to the mechanism of the reaction or to specific interactions with amino acids in the active pocket of the enzyme. Enzymes usually react only with certain types of substrates, whereas chemical reagents are typically only specific to a functional group,^44^ so that inferring information about important substructures in known substrates is especially relevant for biocatalytic transformations.

### Queries on a Template Tree

**Figure 4:**
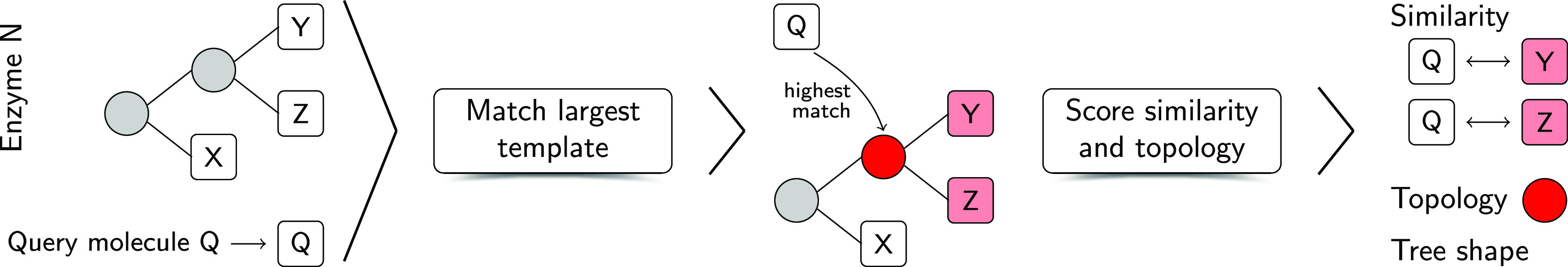
To score the probability of whether a query molecule Q can be processed by enzyme N, the respective template tree is loaded and Q is transformed to a list of possible imaginary transition structures (white squares, only one possibility shown). The ITS of Q is then iteratively matched against the templates (gray circles) in the tree until the most specific (furthest to the right) match is found, highlighted in red. The score then arises from various comparisons of Q to known substrates in the current branch (Y and Z), as well as the location of the template within the tree and the overall shape and diversity of the tree.

Figure 4 schematically depicts how a new substrate or reaction is queried and scored on an extended Hasse diagram. There are three modes to score a reaction or substrate on a given template tree. First, if the Hasse diagram was produced in reaction mode, one can input a reaction SMILES (preferably atom-mapped, else automated mapping via RDT), *i.e.*, one specific reaction, to obtain a score whether the given enzyme will catalyze the query reaction. If the reaction center of the query never occurs in the template tree, the score is zero; otherwise, it is calculated as specified later in this article. Second, one can use a single substrate in SMILES format as a query for a Hasse diagram produced in reaction mode. In this case, the substrate is matched to the reactant fragments of the minimal template in the tree. If a match is found, EHreact identifies whether cosubstrates are missing (for nonunimolecular reactions), as well as whether a transformation can occur in different parts of the molecule, and calculates all possible products of the transformation. For a unimolecular reaction with only one possible product, the substrate is transformed to the corresponding reaction ITS and scored. For possibly regioselective reactions, *i.e.*, different possible products, each possibility is translated to an ITS and scored individually. For each missing cosubstrate in nonunimolecular reactions, the algorithm detects what type of cosubstrate is necessary (for example, an amine donor in an amine transfer reaction, if the given substrate is an amine acceptor) and creates a reaction for each cosubstrate that occurs in the tree, creating one or multiple possible ITSs, which are each scored individually. Third, one can specify one or multiple substrates in SMILES format and omit the cosubstrate search, so that the score is zero if one or more cosubstrates are missing. Multiple possible products due to regioselectivity are detected, and each reaction is scored individually. This mode is beneficial for nonunimolecular reactions if all reactants are known, and thus no cosubstrate search is necessary.

If the Hasse diagram was produced in single-substrate mode, it can only be queried by a single substrate. In this case, the product after transformation of the query substrate remains unknown. If the first substructure in the diagram (the seed) occurs in more than one location in the query, multiple scores are calculated, so that this mode still provides some measure of regioselectivity but it cannot propose cosubstrates or identify the product of a transformation. This functionality is only recommended for a quick scoring of related substrates if the products are not known; for all other cases, we recommend training in reaction mode, so that the full capabilities of EHreact can be exploited during querying and scoring a new substrate or reaction.

### Scoring Function

EHreact scores are calculated as
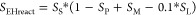
1where *S*_S_ is the maximum Tanimoto similarity of Morgan fingerprints (radius 2, no features) between the query substrate and the known substrates within the current branch. *S*_P_ is the average Tanimoto similarity between all pairs of substrates in the template tree and is a measure of enzyme specificity. 1 – *S*_P_ is thus a measure of enzyme promiscuity. *S*_P_ was capped at 0.8 for practical reasons, i.e., not setting the promiscuity to zero for linear template trees with only a single substrate. *S*_M_ is the mean Tanimoto similarity between the query and all known substrates (within the whole tree, not only the current branch). A larger *S*_P_ (more specific enzyme) necessitates a larger *S*_M_ value to still yield a good overall score. Thus, the difference between *S*_P_ and *S*_M_ is either positive (increasing the overall score) if the query substrate is more similar to the known substrates than the specificity of the enzyme demands or negative (decreasing the overall score) otherwise. *S*_S_, *S*_P_, and *S*_M_ are calculated on the reactants in single-substrate mode or averaged over reactants and products in reaction mode. *S*_L_ scores the position of the highest applicable template within the tree by counting the minimum number of edges to the closest leaf node, where *S*_L_ is calculated as the minimum distance capped at 5, minus 1 so that it equals zero in the ideal case of only one edge to the closest leaf node. Since the range of *S*_L_ is thus much larger than the ranges of *S*_S_, *S*_P_, and *S*_M_, its coefficient is smaller. The coefficients −1, 1, and −0.1 were determined empirically on part of the data. We note that there are several different ways to calculate a score from *S*_S_, *S*_P_, *S*_M_, and *S*_L_, as well as extract other metrics from the template tree. Various scoring schemes and metrics were evaluated during the course of this study, where eq 1 was found to have good performance and generalization qualities. The score is easily customizable in EHreact.

In this work, we compare EHreact scores against simpler similarity scores, where only the maximum Tanimoto similarity between Morgan fingerprints (length 2048, radius 2, no features) to all known reactions is taken into account, similar to ref (23, 24), as well as ref (22), which uses a different fingerprint, though. Different similarity metrics, fingerprint radii, and fingerprints with/without features were tested for their ability to discern between active/inactive substrates (see the Supporting Information), and the chosen metric and parameters performed best. We note that *S*_S_ is not always the same as such a simple similarity score because *S*_S_ is the maximum similarity over the known substrates within the specific branch in the diagram, whereas the latter is usually the maximum similarity over all substrates.

Other scoring schemes in the literature involve combining a similarity score with a further score, for example, a “biological” score in ref (20), which incorporates a cluster analysis of enzymes, as well as the radius at which the rule was extracted. The novelty of our approach is that the difference between *S*_P_ and *S*_M_ characterizes the promiscuity of the enzyme in relation to the observed similarity, which is substantially different from the cluster analysis of ref (20), which characterizes sequence availability. Furthermore, counting the number of steps to the nearest leaf node, *S*_L_, instead of counting the number of steps from the most general rule to the current rule (radius of the rule) provides an advantage when differently sized substrates are known for an enzyme. Namely, scoring via the radius of a rule disadvantages small known substrates versus larger ones since any change in a small substrate will substantially decrease the radius of the applicable rule, even if furthest away from the reaction center.

### Data Preparation

For validation of the scoring function, a set of experimental studies on the substrate ranges of various enzymes were extracted from the literature manually,^45−52^ as well as a study on organic coupling reactions to test the performance of EHreact on organic, nonenzymatic reactions.^53^ Each study reported either the yield or activity of an enzyme/catalyst on a specified substrate under reaction conditions consistent throughout each study. Each reaction was classified as active or nonactive by assigning a threshold manually to yield approximately 10–40% active reactions per data set (thresholds are listed in Table 1); data is available in the Github repository. Since the distribution of yields and activities varied largely between data sets, the thresholds were roughly chosen around the mean of the distribution of each data set plus 1 standard deviation, but comparable results were obtained with different thresholds. For the organic coupling reactions, a larger threshold was chosen to limit the number of active reactions due to the size of the data set. Substrates with unknown products were omitted, as well as enzymes for which no substrate was labeled as active. The number of remaining substrates and enzymes is also listed in Table 1. All reactions were atom-mapped via RDT and corrected by running EHreact on all reactions per class, flagging reactions with a deviant reaction center and correcting the atom-mapping manually. The number of initially correct and incorrect atom-mappings is available in the Supporting Information. A full list of all employed enzymes/reaction classes, including identifiers as used in the respective references, is available in the Supporting Information.

**Table 1:** Summary of Employed Experimental Data (Reference, Number of Substrates, Number of Enzymes/Reaction Classes, and Threshold into Active/Nonactive (Active If > Threshold)

	ref	#*S*	#*E*	thresh.
nitrilases	[^45^]	38	7	50% yield
aminedehydrogenases	[^46^]	18	12	50 mU/mg
alcoholdehydrogenases	[^47^]	65	2	100 nmol/(min·mg)
carboxyl-methyltransferases	[^48^]	17	3	50 pkat/mg
transaminases	[^49^]	10	12	95% yield
tryptophansynthases	[^50^]	9	42	50% yield
amidinotransferases	[^51^]	42	1	30% yield
dehalogenases	[^52^]	46	1	30 (mM·s)^−1^
C(sp^2^)–C(sp^3^) couplings	[^53^]	52–117	7	70% yield

To count the number of reported reactions for various EC classes and enzymes, we furthermore analyzed BRENDA,^30^ RetroRules,^26^ and RHEA,^54^ as described in the following.

To recover enzymatic reactions for various EC classes, the BRENDA database was parsed using a text download of the database as exported in December 2019.^30^ To resolve SMILES strings from substrate and product names, BRENDA ligands were also queried generically from the search portal to export Inchi values for various ligands included in the database. This does not cover all ligands. To supplement the ligands recovered from the search tool, all remaining compounds were queried against PubChem and the Opsin name resolver.^55^ For downstream analysis, all reaction entries with unresolved compounds were removed and duplicates in each EC class were filtered. Code, including instructions for downloading necessary files, is available on Github.^56^ The reactions from EC 1.1.*X* (*X* = 1.145, 1.149, 1.209, 1.213, 1.239, 1.265, 1.283, 1.50, 1.6, 1.64, 1.72, 3.2, 3.6, 3.9), EC 2.6.1.*X* (*X* = 1, 12, 14, 15, 18, 2, 27, 28, 36, 39, 40, 42, 44, 5, 51, 57, 64, 73), and EC 4.1.3.42 were atom-mapped via RDT and corrected manually, as described above, to serve as test-cases for cosubstrate proposal, regioselective prediction, and diagram construction.

Reactions from RetroRules (version rr02 based on the MNXref (version rr02 based on the MNXref version 3.0,^57^ compatible with RetroPath2) were determined for each 4-digit enzyme EC number in the forward direction at the lowest rule diameter after removing duplicate reaction entries where RetroRules splits multisubstrate reactions into multiple single-substrate rules.

For RHEA, reaction ids were cross-linked to their respective amino acid sequences in UniProt and SwissProt to determine the number of unique reaction annotations available per enzyme (not EC class).^58^ Since RHEA follows a hierarchical annotation technique, specific reactions are associated with a broader master class of reaction, if appropriate. If an enzyme was annotated both with its specific reaction class and master class, the master class was removed from the analysis to avoid double counting.

## Results and Discussion

### Exemplary Template Tree Construction

**Figure 5:**
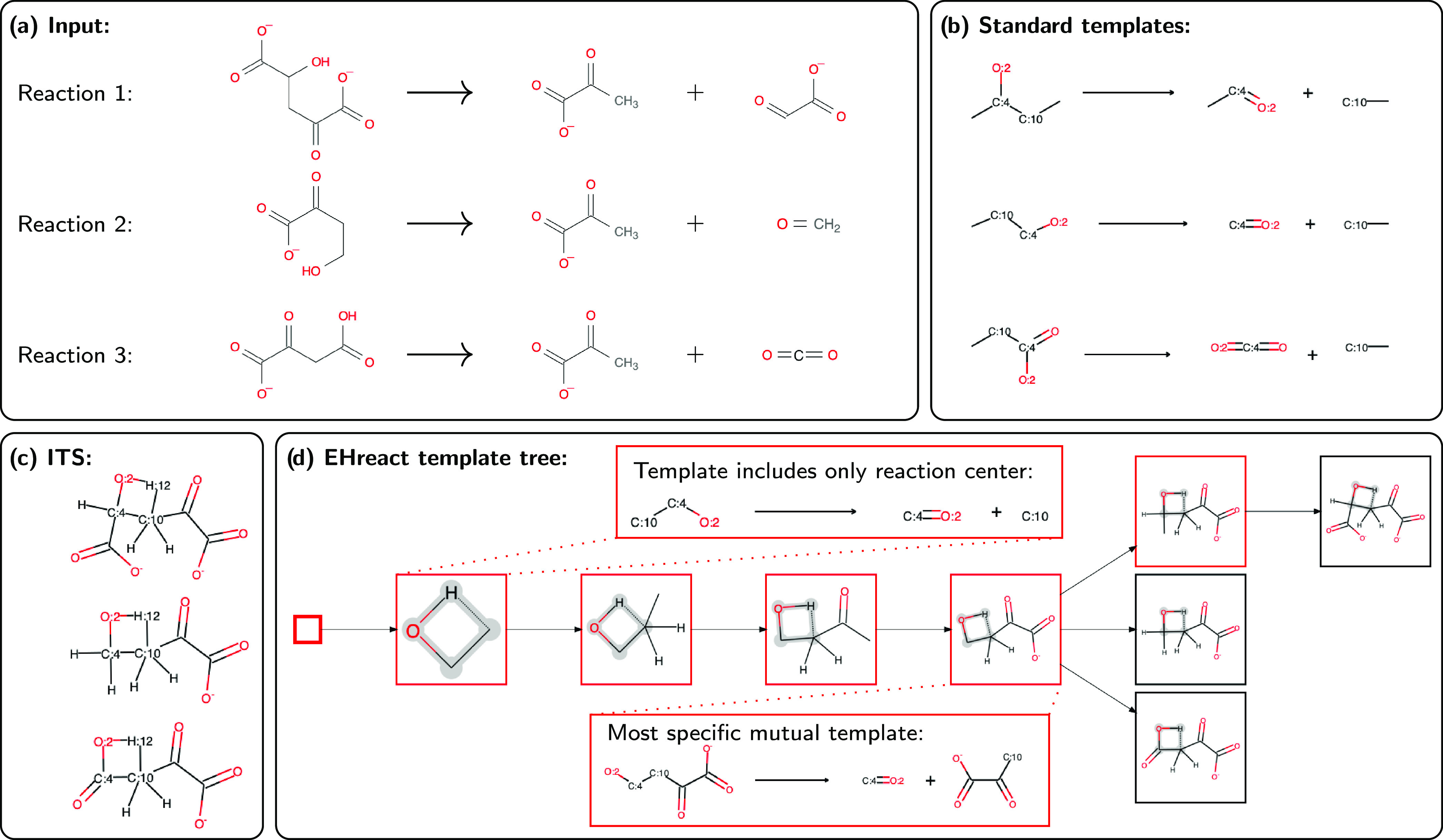
Standard templates and Hasse diagram of three known reactions of 4-hydroxy-2-oxoglutarate lyase (EC 4.1.3.42). Atom-mappings are not shown for atoms that are not in the reaction center. The inputted reactions (a) are transformed to their respective ITSs (c). An iterative substructure search yields the Hasse diagram of all templates (d), which is reprinted as drawn by EHreact (with the two reaction template drawings added). The reaction center is highlighted in gray. Templates (substructures of the ITS pseudo-molecules) are framed in red, and leaf nodes (full ITSs of known reactions) are framed in black. The first template corresponds to the reaction center. The fourth template is the most specific, largest template that describes all inputted reactions and corresponds to the hand-crafted reaction rule for 4-hydroxy-2-oxoglutarate lyase. In contrast, standard template extraction routines (b), here shown for the common choice of including atoms up to one bond away from the reaction center, lead to three different templates, which do not characterize the system well.

To illustrate the transformation of input reactions to their respective imaginary transition structures, as well as the iterative common substructure search around the reaction center, we discuss the enzyme 4-hydroxy-2-oxoglutarate lyase (EC 4.1.3.42), for which BRENDA lists three known substrates, 4-hydroxy-2-oxoglutarate, 4-hydroxy-2-oxobutanoate, and oxaloacetate. The substrates, together with the reaction catalyzed by 4-hydroxy-2-oxoglutarate lyase, are depicted in Figure 5a. The enzyme enables the splitting of a carbon–carbon bond adjacent to a hydroxyl group. The products are for all three cases pyruvate, as well as glyoxylate, formaldehyde, or carbon dioxide, respectively. Extracting reaction templates with literature methods,^26,39^ for example, including all atoms up to one bond away from the reaction center (a common choice), would create three different templates (Figure 5b), which all miss the mutual information inherent to the known reaction. The ITSs of the reactions show a large common substructure (Figure 5c). One side of the substrate is highly conserved, namely, the side that forms pyruvate (everything attached to C:10), while the other side (everything attached to C:4) is diverse in structure and size. This indicates that the pyruvate side is essential for a good fit into the active pocket of the enzyme and/or is involved in the mechanism. And indeed, a mechanistic study on 4-hydroxy-2-oxoglutarate lyase showed specific interactions of the amino acids in the active pocket with the pyruvate side of the substrate, as well as volume restrictions at the same side.^59^ EHreact exploits this mutual information between known reactions by iteratively adding atoms in conserved substructures to the minimum reaction template (first template in Figure 5d). In each step, the algorithm adds only atoms and their corresponding bonds, which are conserved in all reactions and are direct neighbors of current atoms in the template, eventually leading to the fourth template in Figure 5d, which is the most specific template that applies to all input reactions. It identifies that 4-hydroxy-2-oxoglutarate lyase acts on substrates exhibiting the important pyruvate moiety next to the C–C bond to be split and does not specify the other side of the molecule at all, thus corresponding perfectly to a template crafted with expert knowledge of the active pocket and mechanism in this system. Upon further addition of atoms to the template, the diagram splits up into three branches, where two branches lead to leaf nodes directly (the full reaction ITS) and one yields an additional template before ending in a leaf node as well. If the user is interested in a single template, extracting the most specific mutual template (the fourth template in Figure 5d) is sufficient and provides an advantage over traditional template extraction methods where the user decides on an appropriate level of specificity. However, saving the whole template tree and utilizing it in the scoring function were found to be highly beneficial, as demonstrated later in this article. Additional examples of Hasse diagrams (for the BRENDA entry EC 1.1.3.2 and the UniProt entry P23525) are available in the Supporting Information, as well as benchmarks of constructing a diagram and computing the score of a query reaction.

In general, the presented template tree extraction procedure can be useful in a number of scenarios. For example, EHreact reaction mode can be used to extract the single, most specific but mutually applicable template for a set of reactions. Furthermore, calculating a Hasse diagram in single-substrate mode helps to quickly gain an overview over a set of molecules and their common substructures and similarities. A further possible application of EHreact is the reduction of the number of extracted templates from reaction databases for enzymatic and organic reactions alike without losing generality, just as demonstrated in Figure 5, where EHreact yields a single template for all reactions instead of the three different templates as extracted by other routines. It is well known that the number of extracted templates scales with the number of reactions in a database, and a large fraction of templates only occur once even in large data sets.^60^ One could thus reduce the number of templates by using EHreact to extract a template based on common substructures instead of a fixed number of bonds adjacent to the reaction center or possibly even utilize the template tree structure to speed up the application of a template to a molecule, where a missing match to the most general template in a Hasse diagram immediately disqualifies a reaction type, thus making computer-aided synthesis planning easier and faster. EHreact templates should thus be beneficial to retrosynthesis applications since they comprise a small, consistent and mutually exclusive set of templates. The automated selection of generality for each enzyme class allows for a reduced bias toward larger molecules compared to radius-based template extraction. EHreact templates are furthermore balanced and include cofactors and cosubstrates, which makes them applicable to enzymatic cascade design including cofactor recycling, a field of research that currently relies mainly on manually extracted templates.

### Composition of Enzymatic Reaction Databases

The quality of EHreact templates and scoring directly depends on the number of reactions. The number of reactions determines the size and variety of each template tree and thus its ability to create meaningful templates and scores.

**Figure 6:**
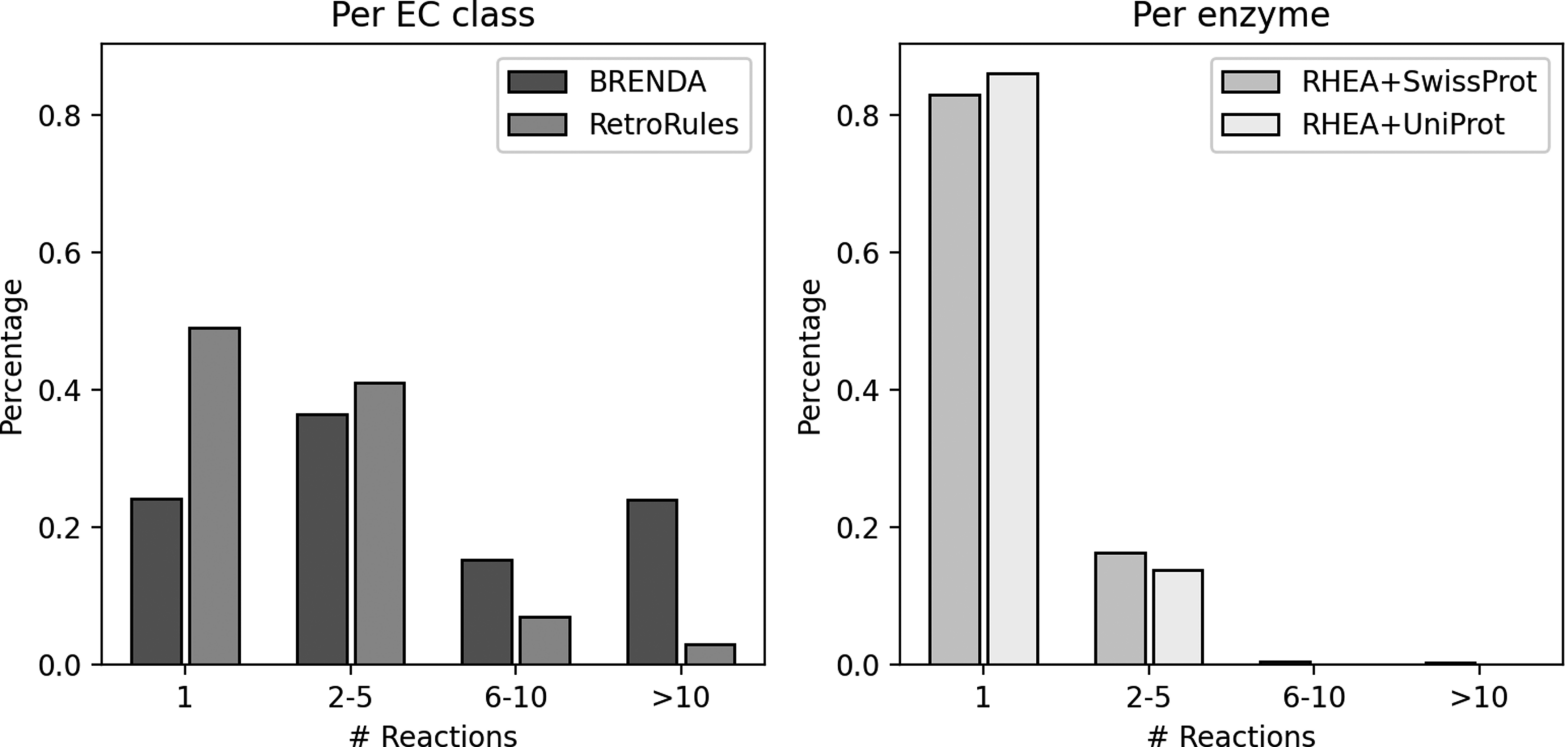
Number of reactions per EC class (left) and per enzyme (right) in different databases.

Figure 6 depicts the number of known reactions per EC class or enzyme in different databases, namely, RHEA (cross-linked with SwissProt and UniProt), BRENDA, and RetroRules. For RHEA, reaction ids were associated with their respective amino acid sequences in UniProt and SwissProt, and unique reaction classes were counted per enzyme. Nearly no differences arose between cross-linking with SwissProt or UniProt, and in the following, we refer to the results from SwissProt only. For BRENDA, unique reactions that had valid entries for reactants and products and could be parsed to SMILES strings were counted for each 4-digit enzyme EC number. If a reaction occurred in both forward and reverse directions in BRENDA, it was only counted once. For RetroRules, the number of reactions per 4-digit enzyme EC number was determined for transformations in the forward direction at the lowest rule diameter after removing duplicate reaction entries where RetroRules splits multisubstrate reactions into multiple single-substrate rules. In total, 76% and 51% of all EC numbers and 17% of all enzymes are associated with more than one reaction for BRENDA, RetroRules, and RHEA, respectively, where EHreact can potentially exploit the mutual information between the reactions. Although there are certainly a number of cases where reported reactions are very dissimilar and thus limit the applicability of EHreact, we believe that template extraction based on sets of reactions is a possible and reasonable choice for enzymatic reactions, especially for databases such as BRENDA, and offers an advantage over the extraction of reaction templates from independent reaction precedents.

### Validation on Experimental Data

We compared the ability of EHreact scores to identify promising substrate/enzyme combinations observed in experimental screening studies against different similarity metrics. Nine recent data sets from the literature were chosen toward this aim, for which both reactants and products were known (opposed to the more prevalent manner of only reporting reactants). Eight studies comprised enzymatic transformations, reporting the activity of different nitrilases,^45^ aminedehydrogenases,^46^ alcoholdehydrogenases,^47^ carboxyl-methyltransferases,^48^ transaminases,^49^ tryptophansynthases,^50^ amidinotransferases,^51^ and dehalogenases^52^ on diverse sets of substrates. An additional study on seven organic C(sp^2^)–C(sp^3^) couplings^53^ was utilized to showcase the performance of EHreact on nonenzymatic transformations.

Leave-one-out experiments were conducted on each data set, where the feasibility of each reaction (substrate/product/enzyme combination) was evaluated by omitting it during the calculation of the template tree (one tree per enzyme) and subsequently calculating a score according to the above-discussed scoring scheme. We calculated scores using both EHreact and a traditional similarity metric (Tanimoto similarity on Morgan fingerprints of length 2048, radius 2, no features; see Supporting Information for other metrics and parameters). Data points labeled as active according to the thresholds in Table 1 were treated as known reactions (inputs) for both EHreact and similarity-based approaches. The area under the curve (AUC) of the receiver operating characteristics was then evaluated per assay (for all leave-one-out experiments), as well as the binary classification accuracy at a threshold of 0.5 (which is close to the mean optimal threshold averaged over all enzymatic systems for both EHreact, 0.43, and similarity scores, 0.58; optimal thresholds for each system are given in the Supporting Information). Other thresholds and F1-scores are available in the Supporting Information, as well as AUC and Acc. for running EHreact in single-substrate mode (instead of reaction mode).

**Figure 7:**
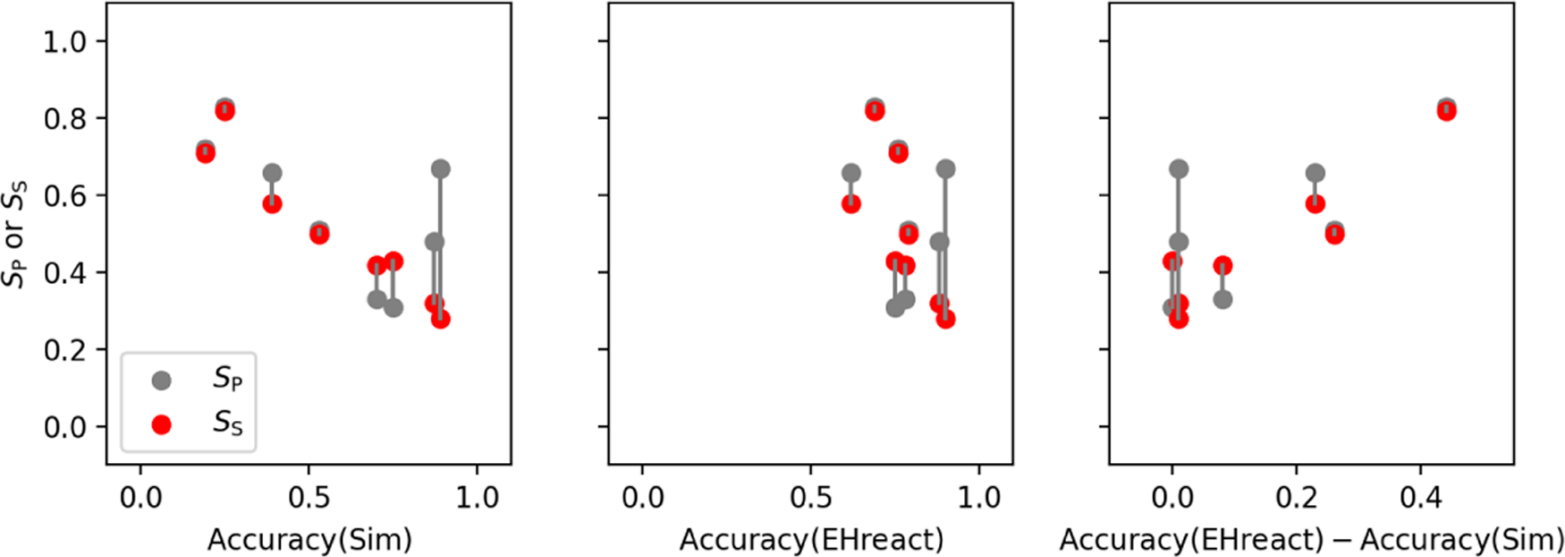
Relationship between the classification accuracy of an assay and the similarity score *S*_S_ and the promiscuity score *S*_P_ calculated via similarity (left), EHreact (center), and their difference (right). The lines connect the respective *S*_S_ and *S*_P_ values of each system. The new EHreact method is much more accurate for nonpromiscuous enzymes or if the new substrate is very similar to substrates in the training set.

Table 2 lists the AUC and accuracy for the classification into active/inactive substrates in reaction mode. In general, EHreact leads to a similar AUC but higher accuracies, with the differences being especially prominent for carboxyl-methyltransferases, transaminases, tryptophansynthases, and amidinotransferases. In these assays, substrates have high similarity scores between each other, but the enzymes only act on a very narrow range on substrates, *i.e.*, are rather selective. In this case, a high similarity score does not necessarily ensure an enzyme being active toward a new substrate. Figure 7 depicts the similarity scores of a new substrate, as well as the similarities between known substrates, over the observed classification accuracies for all eight assays. The classification accuracy of simple similarity metrics significantly decreases with increasing similarity scores (left panel) since the similarity between known substrates also increases, indicating very specific enzymes. Since the specificity/promiscuity of an enzyme is not taken into account, the high similarity scores cause a large number of false positives in the classification. In contrast, the accuracy of EHreact scores (center panel) does not show a dependence on the individual similarities and specificities because they can both contribute to the score (higher specificities necessitate higher similarities to still observe a good overall score). The right panel shows the difference of accuracies via EHreact and similarity scores, which is largest for cases with high individual similarities and specificities. The shortcoming of similarity metrics to discern between specific and promiscuous enzymes was already identified in the literature,^28^ but, to the best of our knowledge, EHreact offers the first systematic scoring scheme to correct for it. This observation is not tied to the threshold used (see the Supporting Information for other thresholds) but a fundamental shortcoming in similarity metrics not discerning between generalist and specialist enzymes and thus necessitating different thresholds for each enzyme. We thus find that the additional information in the shape, size, and diversity of the template tree of known reactions of an enzyme is beneficial for the scoring of new substrates and helps to find a universal scoring threshold across different data sets.

**Table 2:** Area under the Curve AUC and Classification Accuracy Acc. (at a Threshold of 0.5) for Scores Obtained via Similarity or EHreact^a^

	AUC		Acc.	
	Sim.	EHreact	Sim.	EHreact
nitrilases	0.91	**0.93**	0.87	**0.88**
aminedehydrogenases	**0.88**	0.87	0.89	**0.90**
alcoholdehydrogenases	**0.80**	0.77	0.75	**0.75**
carboxyl-methyltransferases	0.82	**0.86**	0.25	**0.69**
transaminases	0.59	**0.73**	0.53	**0.79**
tryptophansynthases	**0.66**	0.57	0.39	**0.62**
amidinotransferases	0.78	**0.82**	0.19	**0.76**
dehalogenases	0.74	**0.76**	0.70	**0.78**
C(sp^2^)–C(sp^3^) couplings	0.70	**0.75**	0.53	**0.75**

a Highest values for AUC and Acc. are printed in bold. Corresponding data for EHreact in single-substrate mode is available in the Supporting Information.

Table 2 furthermore lists classification metrics for a nonenzymatic assay, namely, a set of organic C(sp^2^)–C(sp^3^) coupling reactions, where each name reaction (BF3K-Ni-photoredox, BF3K-Pd-Suzuki, CEC-Ni-Weix, CEC-Ni-photoredox, COOH-Ni-photoredox, MIDA-Pd-Suzuki, and Negishi-Pd) was used to group known reactions, similar to each individual enzyme in the enzymatic assays. Leave-one-out experiments were conducted to score each reaction within each name reaction group. EHreact scores provide an improvement regarding both AUC and accuracy compared to similarity scores, although EHreact scores were developed and tested solely on enzymatic reactions. We expect this improvement to hold for some other organic reactions, too, namely, whenever the structure around the reaction center contributes to the reaction outcome or yield significantly. Although this is certainly not the case for organic reactions in general, it makes EHreact a useful tool for at least some reaction classes.

Next, we investigated whether EHreact still provided an improvement over similarity-based approaches if only a single substrate per enzyme was known. Thus, template trees and similarity comparisons were solely calculated for the most active substrate for each enzyme in each data set, producing linear template trees without any branches. This analysis thus reflects the case of *n* = 1 in Figure 6. In a linear template tree, the promiscuity scores do naturally not come into play, but the location score may still provide a means to penalize modifications close to the reactive center over modifications in other parts of the molecule compared to the reference structure. However, we found no significant trends in the AUC between scores based on similarity and EHreact. For some systems, a penalty based on the location score was beneficial but not for others, indicating that diameter-based template scoring is not necessarily superior to the overall similarity scoring.

### Regioselectivity and Cosubstrate Proposal

**Figure 8:**
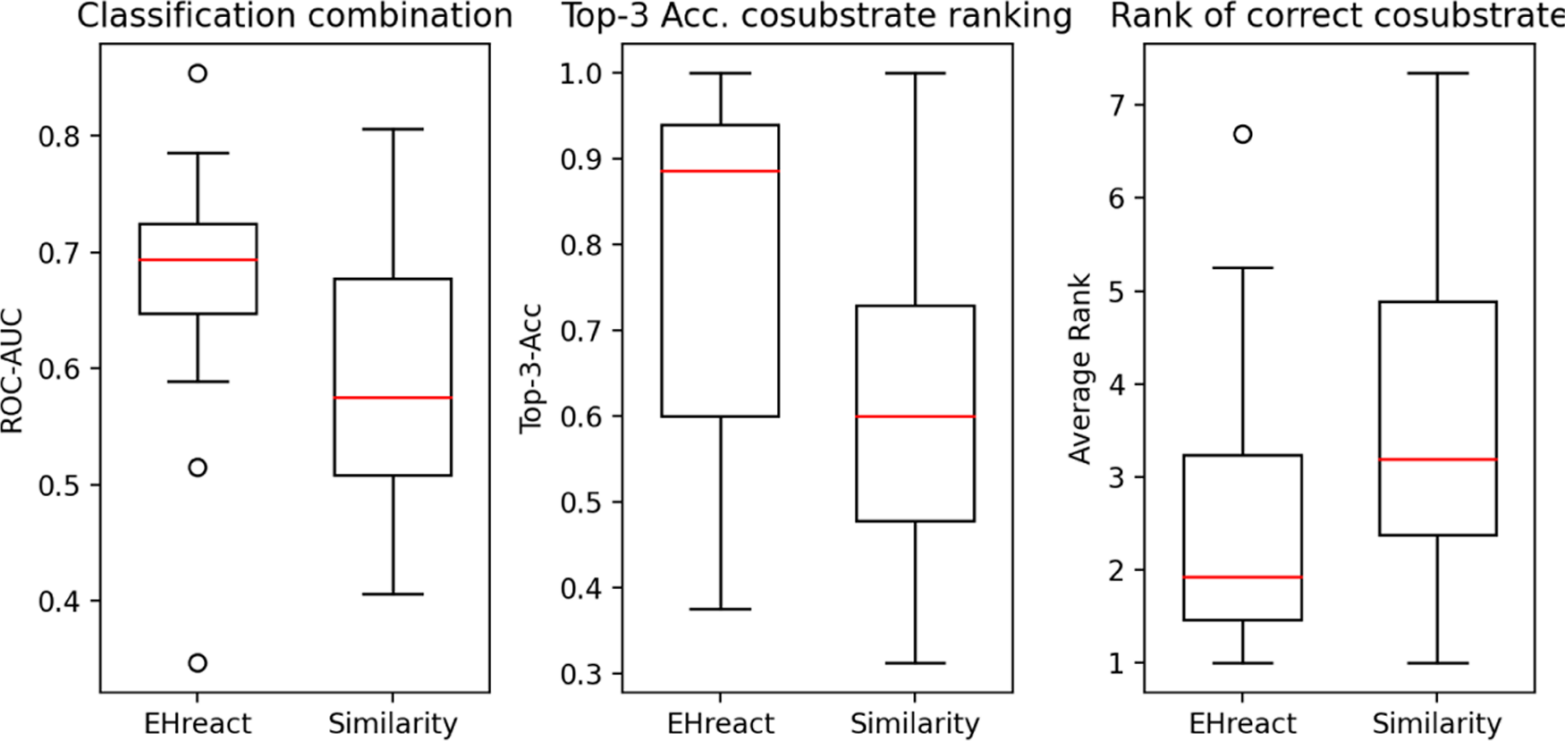
Comparison between EHreact and similarity scores. Boxplots of the ROC–AUC for the classification of whether a combination of substrates is likely (left), top-1-accuracy for the proposal of a cosubstrate (middle), and average rank of the correct cosubstrate (right).

To evaluate the EHreact’s ability to propose meaningful cosubstrates for multisubstrate reactions, we selected EC classes from BRENDA, which report on reactions with two substrates each, have more than 10 known reactions, less than 70% occurrence of the most frequent substrate over all reactions, and molecular weights less than 200 g/mol per substrate. All reactions were then checked for balance, where unbalanced reactions were discarded, and then atom-mapped via RDT. Due to the difficulties of RDT to map some of the reactions, mappings were checked manually and corrected if necessary. This yielded 555 reactions in 18 EC classes, namely, 2.6.1.*X* with *X* = 1, 12, 14, 15, 18, 2, 27, 28, 36, 39, 40, 42, 44, 5, 51, 57, 64, and 73 (transaminase reactions). For the reactions in each EC class, the ability of EHreact and similarity scores to discern between combinations of amine-donors and acceptors as observed in BRENDA (positive data) and all other combinations (obtained by the exhaustive combination of all donors and acceptors within a class corresponding to negative data) was analyzed. We calculated the area under the curve of the receiver-operator-characteristic to obtain a measure of how well the obtained scores can discern between true and artificial combinations of substrates (Figure 8, left panel). EHreact outperforms similarity scores with an average AUC of 0.69 versus 0.59. We furthermore calculated the rank of the correct reaction partner for each substrate, which occurred only once in the reported reactions but its partner occurred in multiple reactions by enumerating all possible reaction partners and calculating scores via EHreact and similarity on the basis of the other known reactions within an EC class. The average ranks are shown in Figure 8, right panel, where EHreact ranks the correct partner on average at rank 2.5 and thus higher than a comparison via similarity (average rank 3.6). The fraction of reactions where the correct partner was identified at rank 1 (top-1-accuracy) is 64% for EHreact and 41% for similarity. Taking into account the first three suggestions (top-3-accuracy), EHreact correctly identifies the cosubstrate in 81% of cases and similarity for 62% of cases. EHreact thus outperforms similarity scores for both classifying whether a given combination of substrates is likely to undergo an enzymatic reaction, as well as ranking suggestions for reaction partners.

Regarding regioselectivity, we selected 13 EC classes from BRENDA where some reactions had multiple possible sites of transformation, here alcohols for oxidoreductase enzymes catalyzing the oxidation of alcohols to ketones/aldehydes (EC 1.1.*X* with *X* = 1.145, 1.149, 1.209, 1.213, 1.239, 1.265, 1.283, 1.50, 1.6, 1.64, 1.72, 3.6, 3.9). We calculated scores for each reaction site using EHreact or similarity scores using the nonregioselective reactions within the same EC class as training reactions. Both EHreact and similarity scores showed 100% top-1-accuracy, thus identifying the correct site of transformation in all cases.

The capability of EHreact to propose and rank cosubstrates, as well as output byproducts, makes it especially attractive for use in the enzymatic cascade design. Designing an efficient cascade includes selecting transformations from a set of possible reactions that recycle cofactors, reduce waste in the form of unwanted byproducts, and find combinations of reactions that drive the equilibrium to the product, which are all tasks that rely on an accurate prediction of cosubstrates, cofactors, and byproducts.

### Limitations

In the following, we briefly summarize the current limitations of our tool since we believe that a critical discussion helps us to prevent unintentional misuse, as well as spark developments and solutions that overcome current shortcomings. Since the software is open source, we furthermore invite interested users to contribute toward this effort.

An apparent limitation of the proposed method is its need for atom-mapped, balanced reactions, which can add additional burden to the preprocessing of databases, where reactions are often unbalanced, and not always atom-mapped, sometimes even incorrectly atom-mapped. In fact, erroneous atom-mappings are a major limitation to all template-based reaction predictions in both organic and biocatalytic syntheses. Incorrect atom-mappings usually cause unique, nonmeaningful ITSs, which branch off at the beginning of the Hasse diagram of templates. EHreact thus provides a framework to easily detect incorrect mappings, but a correction can be tedious and often requires manual interaction. On a similar note, the input of inconsistent configurations, such as open- and closed-loop sugars, leads to an undesired branching in the template diagram. Furthermore, the full functionality of EHreact requires the knowledge of reactants and products for the training set, but substrate screening studies often only report on the reactants but not the products, measuring reaction success by the consumption of the substrate or a cofactor.

We have shown in previous sections that EHreact functions best if more than one reaction per enzyme is known. If only a single reaction is known, the scoring scheme still profits from the multiple templates extracted at different specificities forming a linear template tree in some cases, but if the user wishes to only output a single reaction template, then there is no advantage over other template extraction routines in the literature. For a linear template tree, EHreact cannot determine which specificity or level of generality is best, and the specificity has to be determined by user input (for example, include all atoms up to one bond away from the reaction center, which is the second template in a linear template tree). This only comes into play where the primary use of EHreact is template extraction instead of scoring.

Finally, there are some limitations to the scoring algorithm, too. Although EHreact uses a scoring scheme beyond simple chemical similarity metrics, it is still based on common structures and their similarities. Thus, for enzymatic systems where the activity does not correlate well with conventional molecular descriptors, we also expect that EHreact will not perform well. Other similarity-based approaches will also fail for such cases. Also, an inherent limitation of all similarity-based and structure-based approaches is their inability to extrapolate to new substrates, which are very different from known ones. Although the diversity of the EHreact scoring routine might help us to perform better than a fingerprint similarity comparison for extrapolating to new substrates, we expect its extrapolation ability to be at best mediocre.

## Conclusions

We have introduced a novel method of extracting multiple reaction templates from a set of known reactions and utilizing the mutual information between them to obtain better predictions of the activity of non-natural substrates. The developed open-source software, EHreact, extracts, groups, and saves templates as imaginary transition structures and constructs a Hasse diagram of molecular fragments of the transition states.

EHreact allows for the extraction of single, unique, and mutually exclusive templates at a level of specificity imposed by the set of input reaction, whereas conventional extraction routines lead to multiple, sometimes not mutually exclusive templates and require user-defined criteria of how many atoms to include. Using the most specific mutual template in a Hasse diagram automatically includes all atoms close to the reactive center, which are conserved within the full set of known reactions, without any knowledge about the system. This significantly lowers the number of extracted templates in a database and discerns between specialist and generalist enzymes. It furthermore reduces the bias toward larger molecules that is present in radius-based template sets, thus allowing for predictions of reactions that are missed by current approaches. EHreact can also be used to visualize substrate scopes and specificities of enzymes (or groups of reactions in general) in a straightforward, transparent, and interpretable fashion. It thus offers a white box alternative to black box approaches such as neural network models to predict template specificity for chemical synthesis planning.

The template trees, together with a scoring function, can furthermore be utilized to propose possible transformations on a substrate by a given enzyme, as well as score and rank the proposed reactions according to their anticipated feasibility. The scores allow for a better classification into active and nonactive substrate/enzyme combinations compared to similarity-based scores for experimental screening studies of substrate ranges of diverse enzymes. The scoring scheme was furthermore shown to accurately rank the correct product highest for substrates that can undergo transformations at different positions, as well as correctly propose cosubstrates for multireactant transformations such as amine transfers, which is an important prerequisite for the application in the computer-aided enzymatic cascade design.

We have thus established the extraction and scoring of reaction templates based on Hasse diagrams of common substructures in the imaginary transition structures to be an easy and promising alternative to conventional template extraction and scoring routines, especially where only a few reactions per enzyme are known. We acknowledge that different approaches, such as machine learning of structure–activity relationships of enzymes and substrates, are a very promising alternative for large data sets, with a number of studies published recently.^61,62^ However, for regimes of little data, as presented in this study, we believe that simple heuristic scoring schemes are a more robust and interpretable route toward success and estimate the performance of EHreact to be satisfactory for use in computer-aided pathway design. We plan to utilize EHreact to design multistep synthesis pathways and enzymatic cascades.

## Supporting Information Available

Comparison of the performance of different fingerprints and similarity metrics, tabulated accuracies at different thresholds, results for single-substrate mode, example output of EHreact, further examples of template trees, timing benchmarks, details on the data preparation, and accuracy of RDT atom-mappings (PDF)

## Notes

All data sets used in this study are publicly available. Scripts to parse and preprocess BRENDA are freely available on Github.^56^ The EHreact Python package is freely available on Github.^34^ The repository furthermore contains extensive documentation of the software. This study was executed with default parameters for all analyses. The EHreact repository also contains the preprocessed experimental assay data in CSV format. Further information on the preprocessing of the experimental assay data is given in the Supporting Information. The leave-one-out strategy to create scores for each data point of the assays is described in detail in the main text.
