# Supplementary material for: EHreact: Extended Hasse Diagrams for the Extraction and Scoring of Enzymatic Reaction Templates
Source: J Chem Inf Model. 2021 Sep 29;61(10):4949–61. doi: 10.1021/acs.jcim.1c00921 (PMC8549070; doi:10.1021/acs.jcim.1c00921)
Supplement: Supplementary file 1 — ci1c00921_si_001.pdf [file ci1c00921_si_001.pdf]

Supporting Information: EHreact: Extended Hasse Diagrams for the Extraction and Scoring of Enzymatic Reaction Templates

Esther Heid,[†] Samuel Goldman,[‡] Karthik Sankaranarayanan,[†] Connor W. Coley,[†] Christoph Flamm,[¶] and William H. Green[*,†]

†*Department of Chemical Engineering, Massachusetts Institute of Technology, Cambridge, Massachusetts 02139, United States*

‡*Computational and Systems Biology, Massachusetts Institute of Technology, Cambridge, Massachusetts 02139, United States*

¶*Department of Theoretical Chemistry, University of Vienna, 1090 Vienna, Austria*

E-mail: whgreen@mit.edu

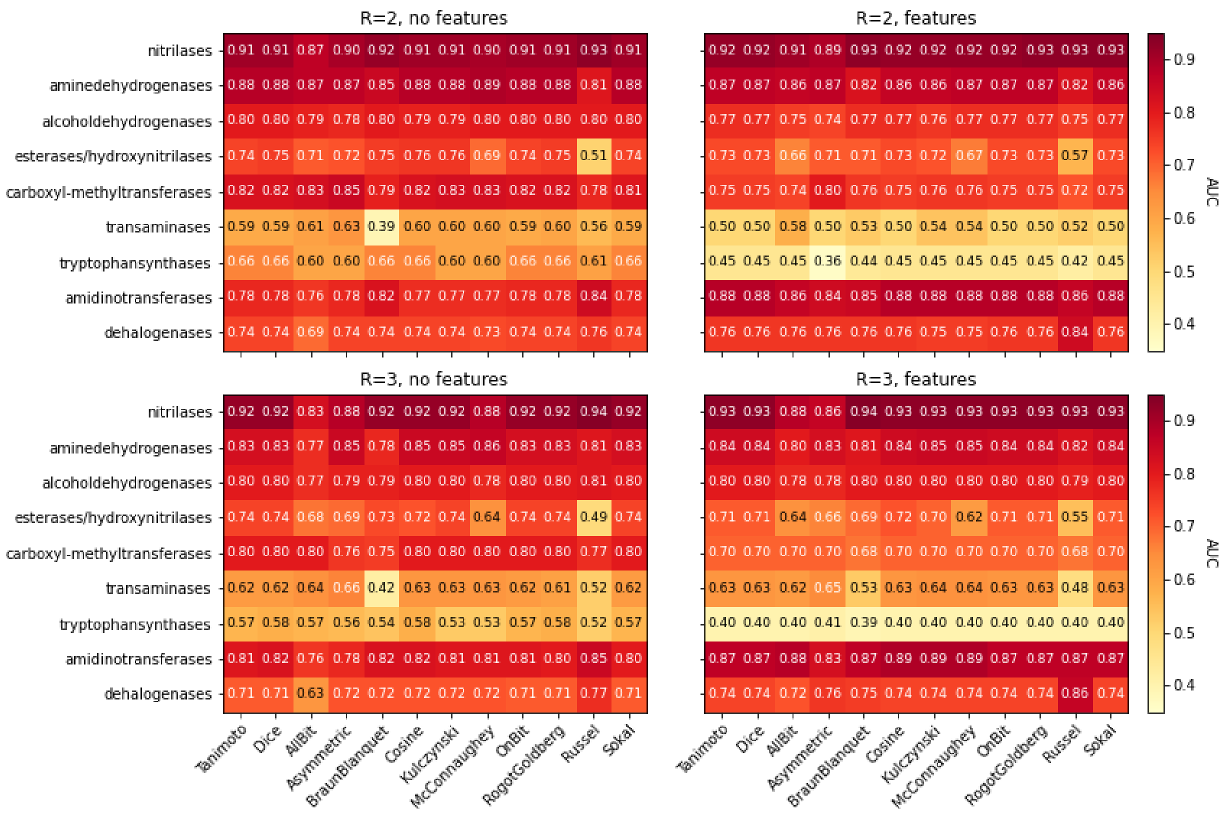

Figure S1: AUC (classification into active/inactive substrates for leave-one-out splits) for Morgan fingerpints at radii 2 and 3, with and without features, for different similarity metrics

S1 Comparison of fingerprints and similarity metrics

Fig. S1 depicts the AUC (for the classification into active/inactive substrates of leave-one-out experiments) for Morgan fingerprints of radius 2 (top) and radius 3 (bottom) with (right) or without (left) features. Twelve similarity metrics, all available in RDKit, were calculated and their performance compared. Fingerprints of radius 2 without features outperform other fingerprints throughout all similarity metrics. Tanimoto, Dice, Cosine, OnBit, RogotGoldberg and Sokal similarity between the fingerprints of a query and the known substrates perform equally well in discerning between active and inactive substrates (and perform better than AllBit, Assymetric, BraunBlanquet, Kulczynski, McConnaughey and Russel similarity). We chose Tanimoto similarity to be used throughout the remainder of this study, since it is routinely applied in several related studies.[S1–S4]

S2 Accuracies at different thresholds

Table S1 lists the classification accuracies at different threshold, Table S2 the corresponding F1 scores. Since the data is imbalanced, a very high threshold creating only negative predictions shows a high accuracy, but the F1 scores clearly label the corresponding classifier as performing badly. In general thresholds around 0.4 and 0.5 lead to good F1 scores and acceptable accuracies in all systems. Optimal thresholds for each system (which maximize the relation between the true positive and false positive rate) are given in Table S3, and differ much between systems for similarity scores.

S3 Single substrate mode

Table S4 lists the *AUC* and *Acc.* for all systems similar to the main article, but with EHreact in single substrate mode (and thus the similarity scores also only taking the reactants into account). Again, similarity and EHreact scores perform equally well for the *AUC* but no uniform threshold applies to all systems, so that the *Acc.* is higher for EHreact.

Table S1: Classification accuracy *Acc.* (at different thresholds t) for scores obtained via similarity or EHreact.

	$t = 0.3$		$t = 0.4$		$t = 0.5$		$t = 0.6$		$t = 0.7$	
	Sim.	EHr.	Sim.	EHr.	Sim.	EHr.	Sim.	EHr.	Sim.	EHr.
Nitrilases	0.62	0.84	0.83	0.89	0.87	0.88	0.87	0.88	0.87	0.87
Aminedehydrogenases	0.72	0.86	0.90	0.89	0.89	0.90	0.90	0.90	0.90	0.90
Alcoholdehydrogenases	0.45	0.52	0.61	0.61	0.75	0.75	0.79	0.78	0.75	0.75
Carboxyl-methyltransf.	0.25	0.31	0.25	0.43	0.25	0.69	0.27	0.82	0.31	0.82
Transaminases	0.36	0.55	0.47	0.69	0.53	0.79	0.51	0.62	0.61	0.63
Tryptophansynthases	0.26	0.39	0.26	0.58	0.39	0.62	0.61	0.61	0.76	0.72
Amidinotransferases	0.17	0.40	0.17	0.57	0.19	0.76	0.38	0.79	0.67	0.83
Dehalogenases	0.48	0.65	0.61	0.74	0.70	0.78	0.74	0.78	0.78	0.78
$C(sp^2)$-$C(sp^3)$ couplings	0.52	0.57	0.53	0.68	0.53	0.75	0.51	0.81	0.74	0.85

Table S2: F1 scores (at different thresholds t) for scores obtained via similarity or EHreact.

	$t = 0.3$		$t = 0.4$		$t = 0.5$		$t = 0.6$		$t = 0.7$	
	Sim.	EHr.	Sim.	EHr.	Sim.	EHr.	Sim.	EHr.	Sim.	EHr.
Nitrilases	0.40	0.59	0.57	0.66	0.51	0.43	0.00	0.11	0.00	0.00
Aminedehydrogenases	0.40	0.45	0.64	0.40	0.15	0.16	0.00	0.00	0.00	0.00
Alcoholdehydrogenases	0.47	0.50	0.52	0.50	0.55	0.53	0.47	0.41	0.11	0.11
Carboxyl-methyltransf.	0.41	0.43	0.41	0.47	0.41	0.62	0.41	0.64	0.43	0.64
Transaminases	0.53	0.58	0.58	0.58	0.59	0.65	0.00	0.00	0.00	0.00
Tryptophansynthases	0.41	0.35	0.41	0.43	0.35	0.43	0.45	0.41	0.15	0.25
Amidinotransferases	0.29	0.36	0.29	0.40	0.29	0.55	0.35	0.40	0.50	0.46
Dehalogenases	0.45	0.56	0.53	0.60	0.56	0.58	0.50	0.50	0.50	0.44
$C(sp^2)$-$C(sp^3)$ couplings	0.37	0.36	0.37	0.38	0.37	0.33	0.26	0.24	0.32	0.26

Table S3: Thresholds which optimize the relation between true positive and false positive rate for scores obtained via similarity or EHreact.

	threshold	
	Sim.	EHreact
Nitrilases	0.48	0.28
Aminedehydrogenases	0.39	0.18
Alcoholdehydrogenases	0.43	0.46
Carboxyl-methyltransf.	0.84	0.57
Transaminases	0.55	0.47
Tryptophansynthases	0.68	0.46
Amidinotransferases	0.71	0.54
Dehalogenases	0.53	0.44
Average	0.58	0.43

Table S4: Area under the curve AUC and classification accuracy $Acc.$ (at a threshold of 0.5) for scores obtained via similarity or EHreact in single substrate mode.

	AUC		$Acc.$	
	Sim.	EHreact	Sim.	EHreact
Nitrilases	0.91	0.93	0.86	0.88
Aminedehydrogenases	0.88	0.87	0.89	0.90
Alcoholdehydrogenases	0.80	0.78	0.76	0.76
Carboxyl-methyltransferases	0.82	0.83	0.69	0.76
Transaminases	0.44	0.70	0.60	0.62
Tryptophansynthases	0.66	0.57	0.74	0.66
Amidinotransferases	0.93	0.92	0.79	0.83
Dehalogenases	0.74	0.72	0.74	0.78

S4 Example output of EHreact

Fig. S2 and Fig. S3 depicts a sample output of EHreact in reaction and single substrate mode. If the computed Hasse diagram is saved to file, it can be loaded via the python package pickle. It consists of the diagram, where each node in the diagram must have a single parent node specified, and can have children nodes specified if it is not a leaf node (not a full pseudo-molecule). Each node furthermore saves an RDKit molecule object of the current template, a list of leaf nodes attached to the node at arbitrary distances, the minimum number of edges to a leaf node and the seed it initially emerged from, together with a few other, less important properties specified in the EHreact documentation online. Each leaf node additionally saves molecular fingerprints of the reactants and products, needed later for scoring. The empty root node of a diagram saves information on the overall similarity of all leaf nodes, as well as a dictionary of atom and bond changes and a list of fragments in case of multi-reactant reactions, which is needed to identify the need of a co-substrate for a reaction, and propose the most promising one.

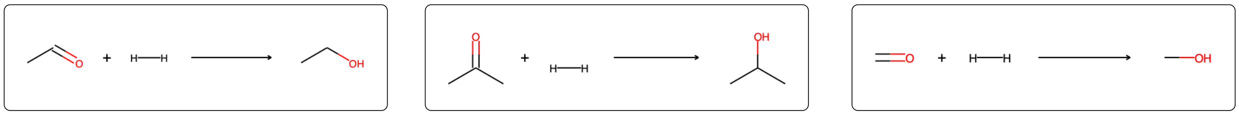

Reaction mode:

Text-based output:

```
 ... root to [C:5]=[O:7].[H:8][H:9] >> [C:5]([O:7][H:8])[H:9]
... [C:5]=[O:7].[H:8][H:9] >> [C:5]([O:7][H:8])[H:9] to [H:8][H:9].[H][C:5](C)=[O:7] >> [H][C:5](C)([O:7][H:8])[H:9]
... [H:8][H:9].[H][C:5](C)=[O:7] >> [H][C:5](C)([O:7][H:8])[H:9] to
[C:1]([H:2])([H:3])([H:4])[C:5]([H:6])=[O:7].[H:8][H:9] >> [C:1]([H:2])([H:3])([H:4])[C:5]([H:6])([O:7][H:8])[H:9]
... [C:5]=[O:7].[H:8][H:9] >> [C:5]([O:7][H:8])[H:9] to C[C:5](C)=[O:7].[H:8][H:9] >> C[C:5](C)([O:7][H:8])[H:9]
... C[C:5](C)=[O:7].[H:8][H:9] >> C[C:5](C)([O:7][H:8])[H:9] to
[C:1]([H:2])([H:3])([H:4])[C:5]([C:6]([H:7])([H:8])[H:9])=[O:10].[H:11][H:12] >>
[C:1]([H:2])([H:3])([H:4])[C:5]([C:6]([H:7])([H:8])[H:9])([O:10][H:11])[H:12]
... [C:5]=[O:7].[H:8][H:9] >> [C:5]([O:7][H:8])[H:9] to [H:1][C:2]([H:3])=[O:4].[H:5][H:6] >>
[H:1][C:2]([H:3])([O:4][H:5])[H:6]
```

Saved python class and example attributes

```
 >>> import pickle
>>> with open('diagram_reaction.pickle','rb') as f:
>>>    d=pickle.load(f)
>>> d
<ehreact.diagram.diagram.Diagram>
>>> d.nodes.keys()
dict_keys(['', '[C:5]=[O:7].[H:8][H:9]>>[C:5]([O:7][H:8])[H:9]',
' [H:8][H:9].[H][C:5](C)=[O:7]>>[H][C:5](C)([O:7][H:8])[H:9]',
'[C:1]([H:2])([H:3])([H:4])[C:5]([H:6])=[O:7].[H:8][H:9]>>[C:1]([H:2])([H:3])([H:4])[C:5]([H:6])([O:7][H:8])[H:9]',
'C[C:5](C)=[O:7].[H:8][H:9]>>C[C:5](C)([O:7][H:8])[H:9]',
'[C:1]([H:2])([H:3])([H:4])[C:5]([C:6]([H:7])([H:8])[H:9])=[O:10].[H:11][H:12]>>
[C:1]([H:2])([H:3])([H:4])[C:5]([C:6]([H:7])([H:8])[H:9])([O:10][H:11])[H:12]',
'[H:1][C:2]([H:3])=[O:4].[H:5][H:6]>>[H:1][C:2]([H:3])([O:4][H:5])[H:6]'])
>>> d.nodes['[C:5]=[O:7].[H:8][H:9]>>[C:5]([O:7][H:8])[H:9]'].rule
<rdkit.Chem.rdchem.Mol>
>>> d.nodes['[C:5]=[O:7].[H:8][H:9]>>[C:5]([O:7][H:8])[H:9]'].is_leaf
False
>>> len(d.nodes['[C:5]=[O:7].[H:8][H:9]>>[C:5]([O:7][H:8])[H:9]'].edges_to_child)
3
```

Figure-based output:

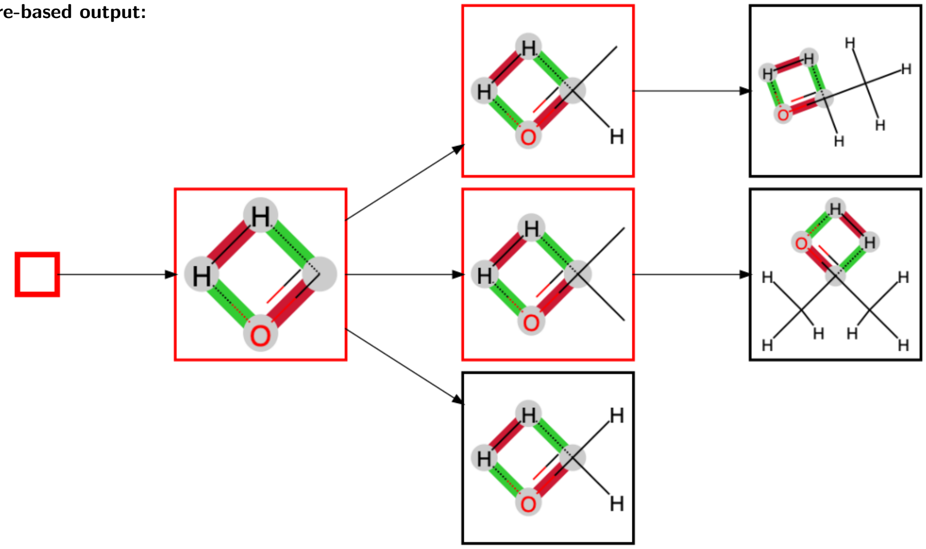

Figure S2: Example EHreact output in reaction mode for the three reactions shown at the top. The text is always produced, whereas saving to a python class and producing a PNG is optional. The python section provides an example of how to load and use the diagram.

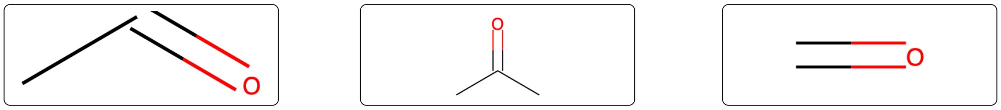

Single substrate mode:

Text-based output:

```
 ... root to C=O
... C=O to [H]C(C)=O
...  [H]C(C)=O to [H]C(=O)C([H])([H])[H]
... C=O to CC(C)=O
... CC(C)=O to [H]C([H])([H])C(=O)C([H])([H])[H]
... C=O to [H]C([H])=O
```

Saved python class and example attributes:

```
 >>> import pickle
>>> with open('diagram_single.pickle','rb') as f:
>>>   d=pickle.load(f)
>>> d.mode
'single_reactant'
>>> d.nodes.keys()
dict_keys(['', 'C=O', '[H]C(C)=O', '[H]C(=O)C([H])([H])[H]',
'CC(C)=O', '[H]C([H])([H])C(=O)C([H])([H])[H]', '[H]C([H])=O'])
>>> d.nodes['C=O'].rule
<rdkit.Chem.rdchem.Mol>
>>> d.nodes['C=O'].edges_to_child
[C=O---[H]C(C)=O, C=O---CC(C)=O, C=O---[H]C([H])=O]
>>> d.nodes['C=O'].edges_to_parent
[root---C=O]
>>> d.nodes['C=O'].min_dist_leaf 1
>>> d.nodes['C=O'].all_leafs
['[H]C(=O)C([H])([H])[H]', '[H]C([H])([H])C(=O)C([H])([H])[H]',
'[H]C([H])=O']
```

Figure-based output:

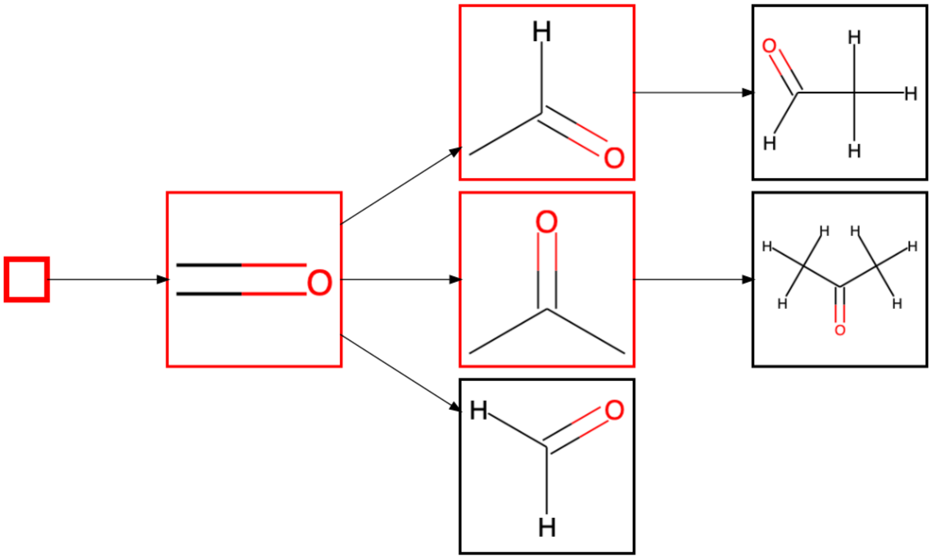

Figure S3: Example EHreact output in single substrate mode for the three substrates shown at the top (and the seed 'C=O'). The text is always produced, whereas saving to a python class and producing a PNG is optional. The python section provides an example of how to load and use the diagram.

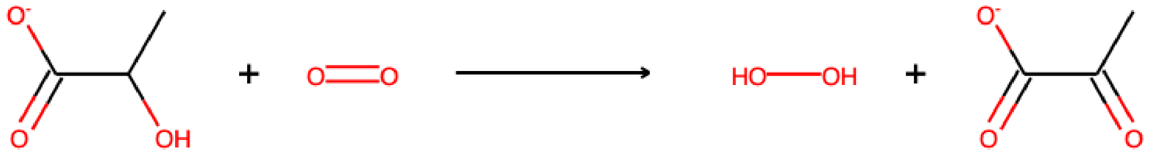

Figure S4: Oxidation of lactate by enzyme EC 1.1.3.2 (stereochemistry omitted)

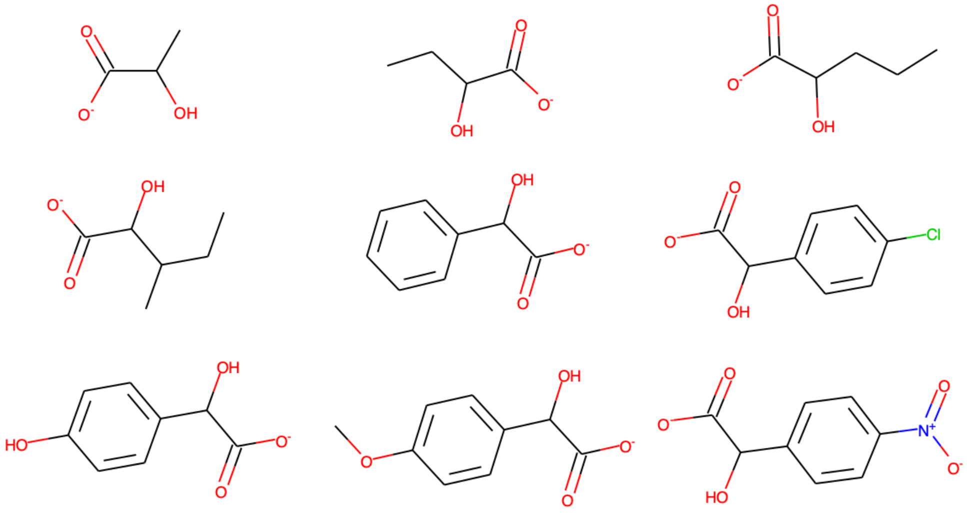

Figure S5: Known substrates of enzyme EC 1.1.3.2 (stereochemistry omitted)

S5 Further template tree demonstration

S5.1 BRENDA EC 1.1.3.2: L-lactate oxidase

L-lactate oxidase (EC 1.1.3.2) catalyzes the oxidation from L-lactate to pyruvate as depicted in Fig. S4. According to BRENDA[S5] other known substrates include modifications on the methyl carbon, such as linear or branched alkyl chains, or aromatic rings which are always substituted in para position, Fig. S5. The Hasse diagram of the known substrates (in transition mode) is depicted in Fig. S6. The structure of the tree depicts exactly the observations made previously, namely that modifications to the primary substrate lactate always occur on the methyl carbon, leaving the carboxyl-group unchanged. Further, the diagram splits

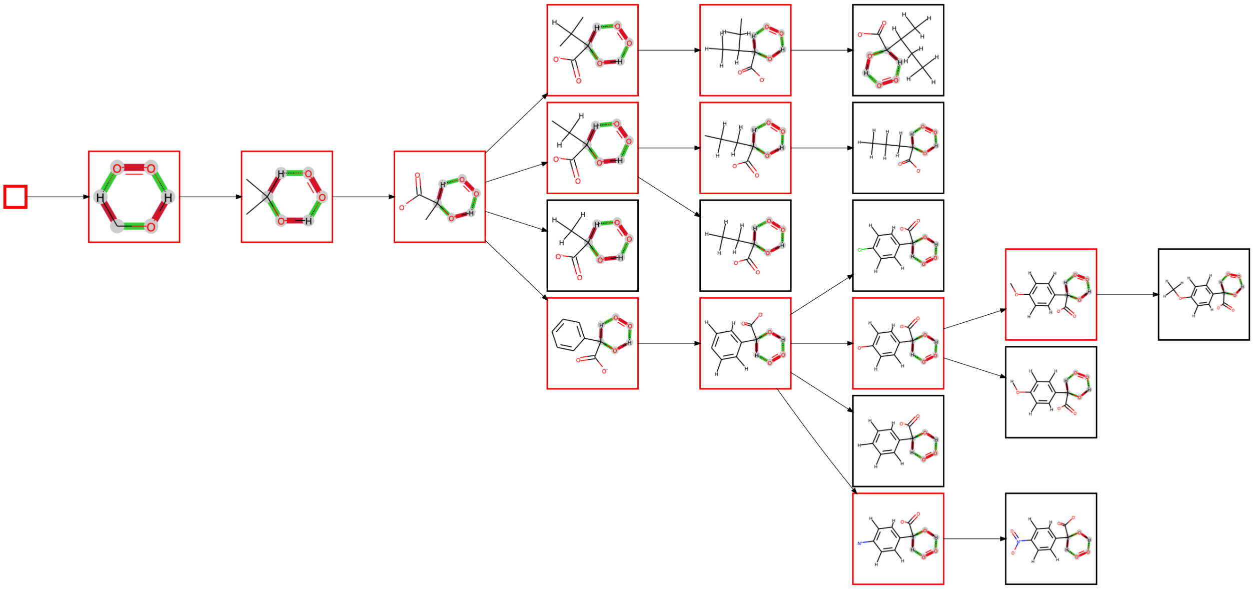

Figure S6: Hasse diagram of the known reactions of enzyme EC 1.1.3.2.

into branched sidechains (first branch from the top), linear sidechains (second branch from the top), no sidechains, which is simply the oxidation of lactate itself (third branch from the top) and aromatic sidechains (bottom branch). The branch with aromatic sidechains furthermore reveals that all known substrates are para-substituted, visible from the two first templates in the branch, the first characterizing that the sidechain is a 6-membered aromatic ring, the second that it is always para-substituted. If a query does not suffice this logic, it is penalized by S_L since the first template (which fits ortho- and meta-substitutions) is at a distance of 2 edges to a leaf node.

The example of lactate oxidase furthermore reveals an advantage of S_L as opposed to a traditional, radius based metric. Fig. S7 depicts two query substrates, one of which is known to be processed according to BRENDA. Both involve a change at a distance of two bonds from the reactive center, and thus only fit standard templates at radius 1. In a radius-based scoring scheme, both queries would be penalized exactly the same. Upon comparison of the templates with the Hasse diagram of the known reactions, however, the first query fits the first three templates, and the distance to the nearest leaf node is 1 ($S_L = 0$), whereas the second query only fits the first two templates, invoking a penalty ($S_L = 1$). In other

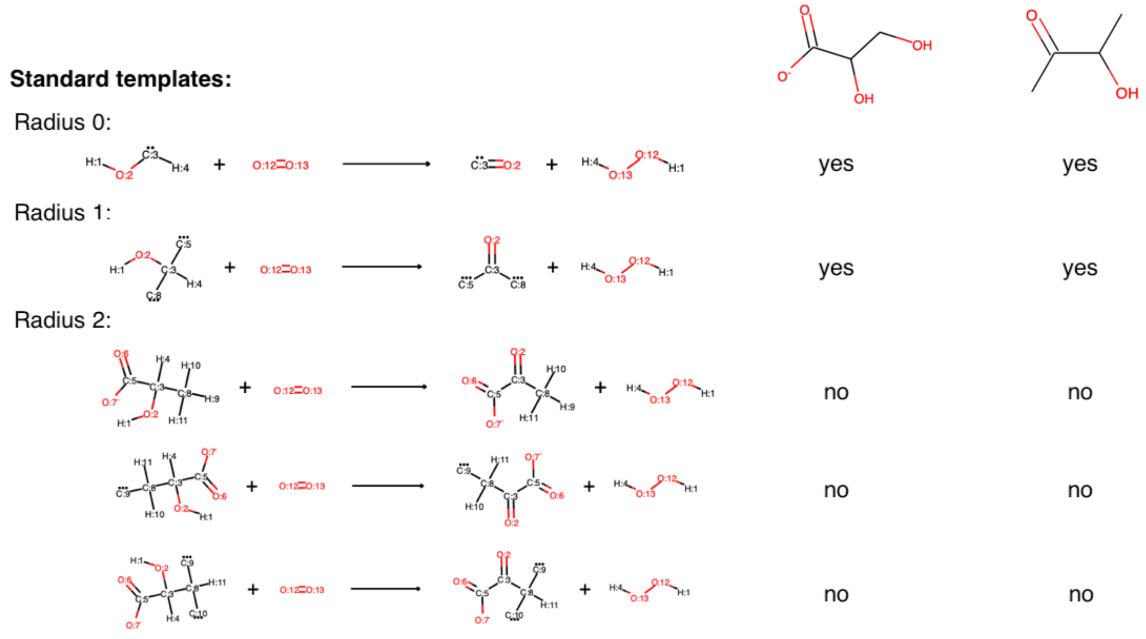

Figure S7: Fit of two query molecules to standard templates extracted at radii 0, 1 and 2 from the nine known reactions.

words, the second query molecule does not feature an important conserved substructure in the known substrates, namely the carboxyl group, and should therefore be scored worse than the first query molecule. The Hasse diagram thus enables a more accurate scoring compared to a set of independent reaction rules extracted at different radii.

S5.2 Uniprot P23525 - 2-methyl-6-phytyl-1,4-hydroquinone methyltransferase

The enzyme P23525 catalyzes an important methylation step in tocopherol and plastoquinone synthesis. Three reactions are reported, see Fig. S8, where three different substrates are methylated by S-adenosyl-L-methionine. The Hasse diagram of these reactions is very large due to the large molecule size, so we instead depict the first eight reaction templates in the diagram (before the diagram splits into branches) in Fig.S9 for the sake of readability. The reaction center is simply a carbon being transferred from sulfur to another carbon (first template), which is specified to be an aromatic carbon in the second template. As the

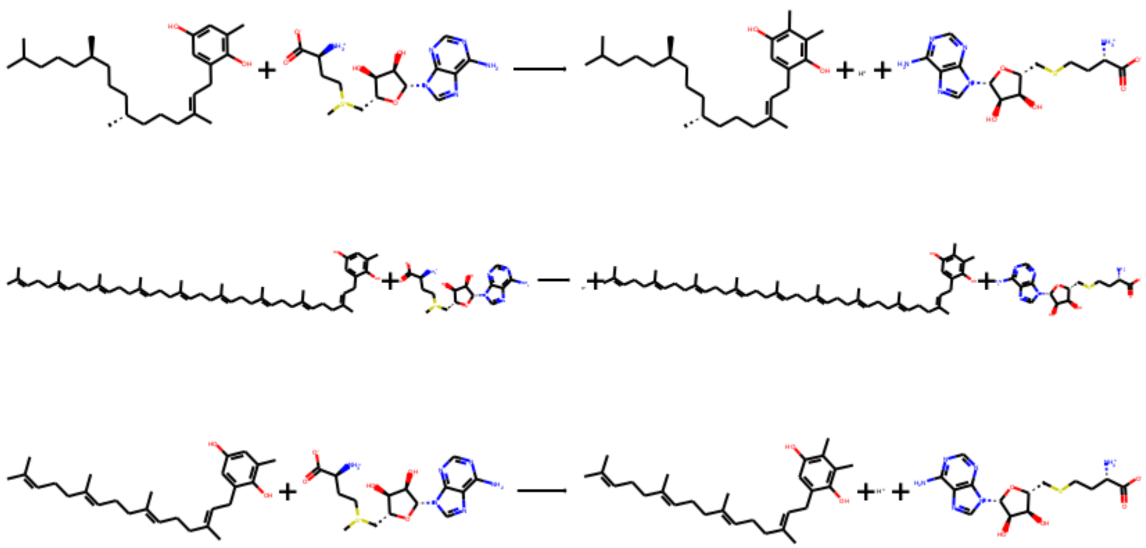

Figure S8: Catalyzed reactions by 2-methyl-6-phytyl-1,4-hydroquinone methyltransferase

templates grow in size, the common substructures, namely S-adenosyl-L-methionine being always the cosubstrate (methyl-donor), as well as the location of the methylation with large conserved substructures in the aromatic ring and its sidechains are identified by the iterative EHreact algorithm. Any change in these conserved structures in the methyl-acceptor, as well as the full methyl-donor molecule cause a penalty in the EHreact score via S_L. This is a major advantage against simple similarity metrics (equal importance of changes throughout the whole molecule) or radius-based similarity metrics (larger importance of changes close to the reaction center). For example, a change in the methyl-donor S-adenosyl-L-methionine is likely to make the reaction unfeasible (according to the reported reactions), whereas variability in the substrate, depending on the location and preferably outside the conserved substructures at the bottom of Fig. S9, may be permissible, although these changes might be closer to the reaction center than changes in the methyl-donor (and thus predicted to be less likely by radius-based metrics).

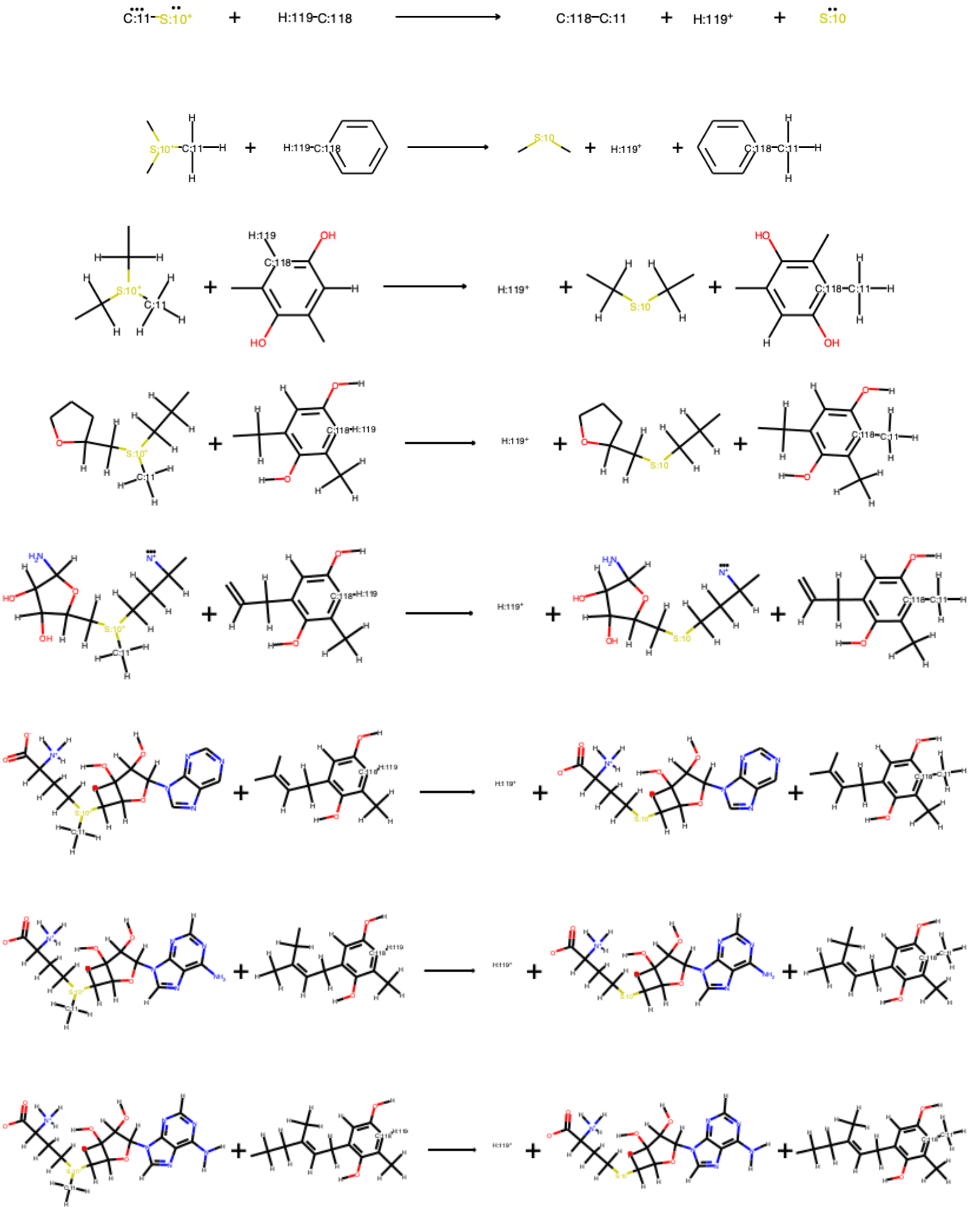

Figure S9: First eight reaction templates in the Hasse diagram of 2-methyl-6-phytyl-1,4-hydroquinone methyltransferase before diagram splits into branches. For readability, atom-map numbers are only shown for the reaction center.

S6 Benchmarking

The time to compute an extended Hasse diagram, as well as the time to predict the score of a given molecule or reaction on a precomputed diagram varies with molecule size and the number of nodes in the tree. The diagrams in Fig. S2 and Fig. S3 were computed in 0.02s (reaction mode) and 0.01s (single substrate mode) on a single CPU on a standard laptop (MacOS 2.2 GHy Quad-Core Intel Core i7). The diagram in Fig. S6 was computed in 0.17s (reaction mode) and 0.07s (single substrate mode) since the diagram consists of more and larger reactions/molecules. The creation of all diagram for the experimental assays in the main article took 84s for a total of 332 diagrams, which is on average 0.25s per diagram (reaction mode). This is slower than the lactate oxidase example since some of the substrates are large, or many substrates are active.

Predicting on the L-lactate oxidase diagram took 0.01s (reaction mode) and 0.001s (single substrate mode) per query reaction/molecule. The scoring of all 1249 substrates from the experimental assays in the main article took 24s, on average 0.02s per query (reaction mode).

We note that the diagram creation and prediction can be parallelized for different enzyme classes or predictions, allowing for fast and convenient processing of multiple systems.

S7 Data details

Table S5 lists the name of each enzyme or system as used in this study and the system name and description as provided in the respective reference.

Table S5: Description of experimental data. "Name" refers to the name of the system within this study (especially the CSV files available on Github), "Description" to the system details in the respective references.

Class	Name	Description
Nitrilases,	E-1	Bradyrhizobium japonicum (Q89GE3) culture 1 (system 3a)
Ref. S6	E-2	Bradyrhizobium japonicum (Q89GE3) culture 2 (system 3b)
	E-3	Bradyrhizobium sp. BTAi1 (A5EKU8) (system 4)
	E-4	Chaetomium globosum (Q2GR86) (system 5)
	E-5	Rhodopseudomonas palustris (Q6N284) culture 2 (system 6b)
	E-6	PRO-NITR010 (system 19)
	E-7	PRO-NITR014 (system 22)
Aminedehydrogenases,	E-1	ChatAmDH with NADH cofactor
Ref. S7	E-2	ChatAmDH with NADPH cofactor
	E-3	IGCAmDH1 with NADH cofactor
	E-4	SgorAmDH with NADH cofactor
	E-5	SgorAmDH with NADPH cofactor
	E-6	IGCAmDH5 with NADH cofactor
	E-7	IGCAmDH5 with NADPH cofactor
	E-8	MATOUAmDH1 with NADH cofactor
	E-9	MATOUAmDH1 with NADPH cofactor
	E-10	MATOUAmDH2 with NADH cofactor
	E-11	MATOUAmDH2 with NADPH cofactor
	E-12	AcolAmDH with NADH cofactor
Alcoholdehydrogenases,	E-1	R-ADH in Tris HCl buffer and 20% hexane
Ref. S8	E-2	S-ADH in Tris HCl buffer and 20% hexane
Carboxyl-methyltransferases,	E-1	Hoya carnosa SAMT
Ref. S9	E-2	Datura wrightii SAMT

Continued on next page

Table S5 – continued from previous page

Class, reference	Name	Description
	E-3	M201H
Transaminases,	E-1	Arthrobacter ArR-ωTA, AlaDH, no cosolvent
Ref. S10	E-2	Arthrobacter ArR-ωTA, AlaDH, DMSO 15% v/v
	E-3	Arthrobacter ArR-ωTA, LDH, no cosolvent
	E-4	Arthrobacter ArR-ωTA, LDH, DMSO 15% v/v
	E-5	Aspergillus terreus AT-ωTA, AlaDH, no cosolvent
	E-6	Aspergillus terreus AT-ωTA, AlaDH, DMSO 15% v/v
	E-7	Aspergillus terreus AT-ωTA, LDH, no cosolvent
	E-8	Aspergillus terreus AT-ωTA, LDH, DMSO 15% v/v
	E-9	Hyphomonas neptunium HN-ωTA, AlaDH, no cosolvent
	E-10	Hyphomonas neptunium HN-ωTA, AlaDH, DMSO 15% v/v
	E-11	Hyphomonas neptunium HN-ωTA, LDH, no cosolvent
	E-12	Hyphomonas neptunium HN-ωTA, LDH, DMSO 15% v/v
Tryptophansynthases,	E-1	Pf2B9
Ref. S11	E-2	TmAzul
	E-3	Tm9D8*
	E-4	TmTriple
	E-5	WT-100-1-B
	E-6	WT-100-1-C
	E-7	WT-100-2-B
	E-8	WT-100-2-C
	E-9	WT-100-2-D
	E-10	WT-100-2-E
	E-11	WT-003-1-B
	E-12	WT-003-1-C
	E-13	WT-003-1-A
	E-14	WT-003-1-D
	E-15	Q90*-003-1-C
	E-16	Q90*-003-1-D

Continued on next page

Table S5 – continued from previous page

Class, reference	Name	Description
	E-17	Q90*-003-1-E
	E-18	Q90*-003-1-A
	E-19	Tri-003-1-D
	E-20	Tri-003-1-E
	E-21	Tri-003-1-F
	E-22	Tri-003-2-B
	E-23	Tri-003-2-C
	E-24	Tri-003-2-D
	E-25	Tri-003-2-E
	E-26	Tri-100-1-B
	E-27	Tri-100-1-C
	E-28	Tri-100-1-D
	E-29	Tri-100-1-G
	E-30	Tri-100-2-C
	E-31	Tri-100-2-D
	E-32	Tri-100-2-E
	E-33	Tri-100-2-F
	E-34	Tri-100-2-A
	E-35	Tri-100-3-B
	E-36	Tri-100-3-C
	E-37	Tri-100-3-D
	E-38	Tri-100-3-E
	E-39	Tri-100-3-F
	E-40	Tri-100-4-D
	E-41	Tri-100-4-E
	E-42	Tri-100-4-F
Amidinotransferases, Ref. S12	SxtG	
Dehalogenases, Ref. S13	LinB	
C(sp^2)-C(sp^3) couplings,	E-1	BF3K-Ni-photoredox

Continued on next page

Table S5 – continued from previous page

Class, reference	Name	Description
Ref. S14	E-2	BF3K-Pd-Suzuki
	E-3	CEC-Ni-Weix
	E-4	CEC-Ni-photoredox
	E-5	COOH-Ni-photoredox
	E-6	MIDA-Pd-Suzuki
	E-7	Negishi-Pd

S8 Correctness of atom-mappings via RDT

Table S6 lists the number of correct and incorrect atom-mappings by RDT for all enzyme classes. Overall, we found 83% atom-mappings to be correct and 17% to be incorrect, where most incorrect mappings stem from either very similar cosubstrates for transaminase reactions or problems assigning the hydrogens for oxidations via NAD(P)H/H^+. When considering only non-hydrogen atoms for determining the accuracy of the mappings, the percentage of correct mappings increases to 95%.

Table S6: Number of correct and incorrect atom mappings via RDT with and without (in brackets) taking hydrogen atoms into account. All wrong mappings were corrected manually.

Class	# correct	# incorrect
Nitrilases	38 (38)	0 (0)
Aminedehydrogenases	0 (18)	18 (0)
Alcoholdehydrogenases	11 (64)	54 (1)
Carboxyl-methyltransferases	17 (17)	0 (0)
Transaminases	10 (10)	0 (0)
Tryptophansynthases	9 (9)	0 (0)
Amidinotransferases	41 (41)	1 (1)
Dehalogenases	46 (46)	0 (0)
EC 1.1.1.145	15 (25)	10 (0)
EC 1.1.1.149	12 (16)	4 (0)
EC 1.1.1.209	13 (18)	5 (0)
EC 1.1.1.213	17 (31)	14 (0)
EC 1.1.1.239	20 (27)	7 (0)
EC 1.1.1.265	16 (16)	0 (0)
EC 1.1.1.283	22 (22)	0 (0)
EC 1.1.1.50	14 (30)	16 (0)
EC 1.1.1.6	18 (19)	1 (0)
EC 1.1.1.64	12 (13)	1 (0)
EC 1.1.1.72	28 (28)	0 (0)

Continued on next page

Table S6 – continued from previous page

Class	# correct	# incorrect
EC 1.1.3.2	10 (10)	0 (0)
EC 1.1.3.6	34 (34)	0 (0)
EC 1.1.3.9	28 (28)	0 (0)
EC 2.6.1.1	15 (15)	5 (5)
EC 2.6.1.12	18 (18)	0 (0)
EC 2.6.1.14	25 (25)	1 (1)
EC 2.6.1.15	34 (34)	0 (0)
EC 2.6.1.18	37 (38)	5 (4)
EC 2.6.1.2	17 (17)	3 (3)
EC 2.6.1.27	13 (13)	3 (3)
EC 2.6.1.28	24 (24)	1 (1)
EC 2.6.1.36	7 (7)	1 (1)
EC 2.6.1.39	13 (13)	4 (4)
EC 2.6.1.40	12 (12)	1 (1)
EC 2.6.1.42	71 (71)	13 (13)
EC 2.6.1.44	28 (28)	0 (0)
EC 2.6.1.5	22 (22)	5 (5)
EC 2.6.1.51	36 (36)	1 (1)
EC 2.6.1.57	50 (50)	8 (8)
EC 2.6.1.64	68 (68)	3 (3)
EC 2.6.1.73	9 (9)	2 (2)
EC 4.1.3.42	3 (3)	0 (0)
all reaction	933 (1063)	187 (57)

References

(S1) Koch, M.; Duigou, T.; Faulon, J.-L. Reinforcement Learning for Bioretrosynthesis. *ACS Synth. Biol.* **2020**, *9*, 157–168.

(S2) Finnigan, W.; Hepworth, L. J.; Flitsch, S. L.; Turner, N. J. RetroBioCat as a Computer-aided Synthesis Planning Tool for Biocatalytic Reactions and Cascades. *Nat. Catal.* **2021**, *4*, 98–104.

(S3) Carbonell, P.; Wong, J.; Swainston, N.; Takano, E.; Turner, N. J.; Scrutton, N. S.; Kell, D. B.; Breitling, R.; Faulon, J.-L. Selenzyme: Enzyme Selection Tool for Pathway Design. *Bioinformatics* **2018**, *34*, 2153–2154.

(S4) Hadadi, N.; Mohammadi Peyhani, H.; Miskovic, L.; Seijo, M.; Hatzimanikatis, V. Enzyme Annotation for Orphan and Novel Reactions Using Knowledge of Substrate Reactive Sites. *P. Natl. Acad. Sci.* **2019**, *116*, 7298–7307.

(S5) Jeske, L.; Placzek, S.; Schomburg, I.; Chang, A.; Schomburg, D. BRENDA in 2019: A European ELIXIR Core Data Resource. *Nucleic Acids Res.* **2019**, *47*, D542–D549, `https://www.brenda-enzymes.org/` (accessed 2019/12/10).

(S6) Black, G. W.; Brown, N. L.; Perry, J. J. B.; Randall, P. D.; Turnbull, G.; Zhang, M. A High-throughput Screening Method for Determining the Substrate Scope of Nitrilases. *Chem. Comm.* **2015**, *51*, 2660–2662.

(S7) Caparco, A. A.; Pelletier, E.; Petit, J. L.; Jouenne, A.; Bommarius, B. R.; de Berardinis, V.; Zaparucha, A.; Champion, J. A.; Bommarius, A. S.; Vergne-Vaxelaire, C. Metagenomic Mining for Amine Dehydrogenase Discovery. *Adv. Synth. Catal.* **2020**, *362*, 1–11.

(S8) Madden, K.; Todd, P. M.; Urata, K.; Russell, A.; Vincent, K.; Reeve, H. A Pharmacophore-based Approach to Demonstrating the Scope of Alcohol Dehydrogenases. *ChemRxiv* **2020**, *Preprint*, 10.26434/chemrxiv.13134767.v1.

(S9) Huang, R.; Hippauf, F.; Rohrbeck, D.; Haustein, M.; Wenke, K.; Feike, J.; Sorrelle, N.; Piechulla, B.; Barkman, T. J. Enzyme Functional Evolution Through Improved Catalysis of Ancestrally Nonpreferred Substrates. *P. Natl. Acad. Sci.* **2012**, *109*, 2966–2971.

(S10) Mutti, F. G.; Fuchs, C. S.; Pressnitz, D.; Sattler, J. H.; Kroutil, W. Stereoselectivity of Four (R)-Selective Transaminases for the Asymmetric Amination of Ketones. *Adv. Synth. Catal.* **2011**, *353*, 3227–3233.

(S11) Rix, G.; Watkins-Dulaney, E. J.; Almhjell, P. J.; Boville, C. E.; Arnold, F. H.; Liu, C. C. Scalable Continuous Evolution for the Generation of Diverse Enzyme Variants Encompassing Promiscuous Activities. *Nat. Commun.* **2020**, *11*, 1–11.

(S12) Lukowski, A. L.; Mallik, L.; Hinze, M. E.; Carlson, B. M.; Ellinwood, D. C.; Pyser, J. B.; Koutmos, M.; Narayan, A. R. Substrate Promiscuity of a Paralytic Shellfish Toxin Amidinotransferase. *ACS Chem. Biol.* **2020**, *15*, 626–631.

(S13) Kmunícek, J.; Hynková, K.; Jedlicka, T.; Nagata, Y.; Negri, A.; Gago, F.; Wade, R. C.; Damborskỳ, J. Quantitative Analysis of Substrate Specificity of Haloalkane Dehalogenase LinB from Sphingomonas Paucimobilis UT26. *Biochemistry* **2005**, *44*, 3390–3401.

(S14) Dombrowski, A. W.; Gesmundo, N. J.; Aguirre, A. L.; Sarris, K. A.; Young, J. M.; Bogdan, A. R.; Martin, M. C.; Gedeon, S.; Wang, Y. Expanding the Medicinal Chemist Toolbox: Comparing Seven C (sp2)–C (sp3) Cross-Coupling Methods by Library Synthesis. *ACS Med. Chem. Lett.* **2020**, *11*, 597–604.
